# Deep learning-based phenotyping of lettuce diseases using Efficient-FBM-FRMNet for precision agriculture

**DOI:** 10.3389/fpls.2025.1704647

**Published:** 2025-11-28

**Authors:** Parul Nasra, Sheifali Gupta, Mudassir Khan, Jaibir Singh, Bayan Alabdullah, Abrar Almjally, Ruby Pant, Nitin Kumar, Salil Bharany

**Affiliations:** 1 Chitkara University Institute of Engineering and Technology, Chitkara University, Rajpura, Punjab, India; 2 Department of Computer Science, College of Computer Science, Applied College Tanumah, King Khalid University, Abha, Saudi Arabia; 3 Jadara University Research Center, Jadara University, Irbid, Jordan; 4 School of Computer Science & Engineering, Galgotias University, Greater Noida, Uttar Pradesh, India; 5 Department of Information Systems, College of Computer and Information Sciences, Princess Nourah bint Abdulrahman University, Riyadh, Saudi Arabia; 6 College of Computer and Information Sciences, Imam Mohammad Ibn Saud Islamic University (IMSIU), Riyadh, Saudi Arabia; 7 Department of Mechanical Engineering, Uttaranchal University, Dehradun, Uttarakhand, India; 8 Department of Mechanical Engineering, Graphic Era (Deemed to be University), Clement Town, Dehradun, India; 9 University Center for Research and Development, Chandigarh University, Mohali, Punjab, India

**Keywords:** lettuce disease detection, deep learning, efficientNetB4, feature bottleneck module (FBM), dilated convolutions, feature refinement module (FRM)

## Abstract

Lettuce (*Lactuca sativa*), a widely cultivated leafy vegetable, is highly susceptible to bacterial and fungal infections that severely reduce yield and quality. Rapid and accurate disease identification is therefore essential for precision agriculture and sustainable crop management. This study proposes Efficient-FBM-FRMNet, a modular deep learning framework for automated lettuce disease detection. The model integrates EfficientNetB4 with dilated convolutions, a Feature Bottleneck Module (FBM) for redundancy reduction, a Reasoning Engine for higher-order semantic inference, and a Feature Refinement Module (FRM) for enhanced generalization. The framework was trained and validated on a publicly available dataset of 2,813 lettuce leaf images (bacterial, fungal, and healthy classes) using stratified 5-fold cross-validation. The proposed Efficient-FBM-FRMNet achieved an overall accuracy of 97.5%, outperforming baseline CNNs such as EfficientNetB4, ResNet50, and DenseNet121. It demonstrated superior precision (96.0%), recall (96.6%), and F1-score (97.0%), confirming its robustness and consistency across multiple folds. Statistical significance analysis (p < 0.05) verified that the performance gains were not due to random variation. The integration of FBM, Reasoning Engine, and FRM enhances discriminative feature learning, interpretability, and stability while reducing computational cost (8.2 MB model size, 23 ms inference). These results demonstrate the model’s potential for real-world deployment in greenhouse monitoring, UAV-based surveillance, and mobile diagnostic systems, contributing to sustainable, AI-driven precision agriculture.

## 1 Introduction

Lettuce (*Lactuca sativa*) is a leafy green vegetable consumed worldwide and an essential component of both subsistence and commercial agriculture. In 2021, global lettuce and chicory production was approximately 27 million tonnes, with China contributing more than 53% of the output. Lettuce plants are highly vulnerable to a variety of diseases, primarily bacterial and fungal, that cause severe yield and quality losses. For instance, *Sclerotinia sclerotiorum* (white mold) and *Alternaria* species are responsible for devastating fungal outbreaks leading to heavy economic losses (Abidi et al., 2024). Other notable diseases include downy mildew (*Bremia lactucae*), bacterial leaf spot (*Xanthomonas campestris*), lettuce mosaic virus, and fusarium wilt (*Fusarium oxysporum* f. sp. *lactucae*), all of which severely affect crop quality and productivity (Nigar et al., 2024; Wu et al., 2025). If undetected, these pathogens manifest as discoloration, wilting, necrotic lesions, and stunted growth, ultimately leading to major agricultural losses. Traditionally, disease identification relies on expert visual inspection, which is time-consuming, error-prone, and infeasible for large-scale monitoring. These limitations underscore the necessity of automated and intelligent disease detection systems for precision agriculture.

With the rising demand for sustainable farming, growers increasingly seek accurate, scalable, and interpretable diagnostic tools. Deep learning–based approaches, especially convolutional neural networks (CNNs), have demonstrated excellent performance in plant disease classification by automatically learning hierarchical features from leaf images (Shoaib et al., 2022; Joseph et al., 2024; Pajany et al., 2024). Unlike manual methods, CNN-based models enable rapid, high-throughput, and objective diagnosis. Their integration with UAVs, greenhouse monitoring systems, and mobile applications can empower farmers to monitor crops in real time, improving early intervention and reducing losses. For lettuce in particular, which is often cultivated under controlled environments such as hydroponics and greenhouses, there is strong motivation to design lightweight yet robust models capable of handling varying lighting, backgrounds, and symptom overlap, while still being interpretable for practical agricultural use.

Despite notable progress, important gaps remain in existing research. First, many CNN and transfer learning approaches rely on fixed receptive fields, which restrict their ability to capture fine-grained and multi-scale lesion patterns unique to lettuce leaves (Desai and Ansari, 2023; Damak et al., 2024). Second, most reported works predominantly focus on maximizing accuracy while neglecting interpretability, leaving it unclear which visual cues drive predictions a limitation that reduces trust and hinders adoption in real-world farming (Yeoh et al., 2023; Husain et al., 2024). Third, existing models often lack explicit mechanisms for feature refinement or redundancy reduction, making them less robust under environmental variations such as inconsistent lighting, occlusions, or overlapping symptoms (Gulzar and Ünal, 2025). Finally, current studies tend to emphasize single, monolithic backbones without exploring modular designs that combine complementary components such as bottlenecking, reasoning, and feature calibration. This gap has limited the development of lightweight architectures that simultaneously achieve high accuracy, robustness, and interpretability suitable for real-time deployment (Maurya et al., 2024; Sudhakar, 2024).

To address these challenges, this work proposes Efficient-FBM-FRMNet, a modular deep learning framework that combines EfficientNetB4 with dilated convolutions, a FBM, a Reasoning Engine, and a FRM. The proposed architecture is designed to overcome the limitations of existing CNN-based lettuce disease detection by capturing multi-scale lesion features, reducing redundancy, modeling higher-order interactions, and refining class-specific cues. Experimental evaluation on a publicly available dataset demonstrates that Efficient-FBM-FRMNet consistently outperforms state-of-the-art models in terms of accuracy, precision, recall, and F1-score, highlighting its potential for practical integration into greenhouse monitoring, UAV-assisted surveillance, and mobile diagnostic platforms.

The contributions are:

- The proposed Efficient-FBM-FRMNet is a deep learning model that combines EfficientNetB4 with dilated convolutions, an FBM (feature bottleneck module), a reasoning engine, and a FRM (feature refinement module) to enhance feature representation, interpretability, and classification robustness for lettuce disease detection.
- The inclusion of dilated convolutions expands the receptive field without increasing parameters, allowing the network to capture both fine-grained lesion textures and large, diffuse disease regions effectively. This enables reliable differentiation between visually similar bacterial and fungal infections.
- The FBM bridges the high-dimensional feature maps and the classifier by compressing redundant representations through convolution, batch normalization, and pooling operations. This process emphasizes critical visual cues such as lesion texture, necrosis, and discoloration while reducing background noise.
- The reasoning engine acts as a semantic inference module that models complex non-linear dependencies among features and refines decision boundaries by mimicking expert-like reasoning between lesion patterns. Unlike conventional dense-layer classifiers, it functions as an interpretable reasoning stage that bridges compact feature embeddings (from FBM) and refined representations (in FRM), enhancing both diagnostic interpretability and classification robustness.
- The FRM serves as the final regularization stage, improving the stability and generalization of learned embeddings under diverse lighting, orientation, and leaf conditions. This refinement strengthens class separability and ensures consistent performance on unseen samples.

The rest of this paper is organized as follows: Section 2 presents the materials and methods, including dataset details, preprocessing techniques, and performance metrics. Section 3 introduces the proposed Eff-FBM-FRMNet architecture and explains its key components. Section 4 discusses the experimental results, analysis, and visual interpretations. Section 5 provides the statistical evaluation and significance testing of the proposed model. Finally, Section 6 concludes the paper and outlines future research directions.

## 2 Literature review

Deep learning has been widely explored for plant disease detection across a variety of crops. For example, an E-CNN integrating InceptionV3 and DenseNet169 was used to diagnose micro-nutrient deficiencies in bananas, analyzing 4,000 images from the Mendeley dataset and achieving 98.62% validation accuracy with an F1-score of 93% (Sudhakar, 2024). Similarly, another study combined pre-trained models such as VGG19, InceptionV3, Xception, DenseNet169, and DenseNet201 with the Mendeley banana dataset (3,450 images), again reporting validation accuracy of 98.62% and an F1-score of 93% (Ashraf et al., 2024). These results underscore the power of ensemble and transfer learning methods in improving agricultural diagnostics.

Researchers have also applied transfer learning to soybean seed classification. The Researchers in (Pillai et al., 2023) developed a DenseNet121 transfer learning system for plant disease classification. They achieved 97.38% accuracy, showing effective disease detection. By training on 5,513 images of five soybean seed classes and applying an adaptive learning rate, models achieved 98.73% accuracy, outperforming traditional methods (Gulzar, 2024). Similarly, CNNs applied to the PlantVillage dataset comprising 54,306 images from 14 crops and 26 diseases achieved 95% training accuracy and 94% testing accuracy (Rs and Sugumar, 2023). In rice, a hybrid CNN-based transfer learning method using pre-trained VGG-16 reached 90.8% validation accuracy on the Rice_Leaf_Dataset, which contained bacterial leaf blight, brown spot, and leaf smut classes (Tunio et al., 2021). These studies demonstrate the applicability of deep learning across a wide range of crop species.

Large-scale datasets such as PlantVillage (54,343 images of 38 plant diseases across 14 crops) have enabled the application of deep architectures like Xception and GoogleNet, which achieved classification accuracy of 98.76% (Arid et al., 2023). Further comparisons of MobileNetV3, InceptionV3, Xception, VGG19, DenseNet121, ResNet101, and EfficientNetB3 found that DenseNet121 and EfficientNetB3 nearly reached perfect accuracy, detecting plant diseases with 98.7% accuracy (Gulzar et al., 2024). In another study, a CNN optimized on an augmented PlantVillage dataset (87,000 images, 38 classes) achieved 98.01% training accuracy and 94.33% testing accuracy (Belmir et al., 2023). These large-scale results confirm the robustness of CNNs and transfer learning under diverse crop conditions.

Several researchers have targeted rice-specific diseases as well. A study focused on rice leaf blight, brown spot, and bacterial blight emphasized precision as a main performance metric and demonstrated the advantage of combining deep learning and machine learning methods (Seelwal et al., 2024). Lettuce-specific research has also gained attention. For instance, a CNN trained on 8,007 images of healthy and diseased lettuce leaves reported 95.62% accuracy, 97.98% precision, 95.29% recall, and an F1-score of 96.61% (Rajendiran et al., 2024). Additionally, the SaudiArabiaFlora dataset introduced a multi-species plant collection, and the proposed MIV-PlantNet model combining MobileNet, Inception, and VGG architectures achieved 99% accuracy, 96% precision, and 98% F1-score (Amri et al., 2024). These contributions extend the range of datasets and validate the effectiveness of hybrid models.

Further studies developed lettuce-specific image catalogs. One dataset categorized lettuce plants into healthy, pest-damaged, and disease-damaged classes, and a CNN achieved 95.72% accuracy, 97.03% precision, and 95.12% recall (Barcenilla and Maderazo, 2023). Other works applied CNNs to different agricultural datasets, such as 21,662 images across four disease classes (Broken, Discolored, Silk Cut, and Pure), using layers like Average Pooling, Flatten, Dense, Dropout, and Softmax for classification (Alkanan and Gulzar, 2024). The most directly relevant lettuce study proposed a compact Conv-7 DCNN, which trained and tested on a public Kaggle dataset of hydroponically grown lettuce leaves, achieving 97% accuracy and outperforming widely used CNN backbones (Rathor et al., 2025).

In summary, while studies (Tunio et al., 2021; Arid et al., 2023; Barcenilla and Maderazo, 2023; Belmir et al., 2023; Pillai et al., 2023; Rs and Sugumar, 2023; Alkanan and Gulzar, 2024; Amri et al., 2024; Ashraf et al., 2024; Gulzar, 2024; Gulzar et al., 2024; Rajendiran et al., 2024; Seelwal et al., 2024; Sudhakar, 2024; Rathor et al., 2025) confirm that CNNs, transfer learning, and hybrid deep learning methods are highly effective in agricultural disease detection, gaps remain. Most architectures rely on fixed receptive fields that limit their ability to capture multi-scale lesion patterns. Additionally, interpretability is rarely addressed, leaving it unclear which features drive predictions. Few approaches incorporate explicit feature refinement or redundancy reduction, which weakens robustness in real-world conditions. Finally, lightweight modular frameworks that combine bottlenecking, reasoning, and refinement are underexplored. These shortcomings highlight the necessity of designing architectures, such as the proposed Efficient-FBM-FRMNet, that not only deliver high accuracy but also improve robustness, interpretability, and deployability for lettuce disease detection.

## 3 Proposed methodology

**Figure 1:**
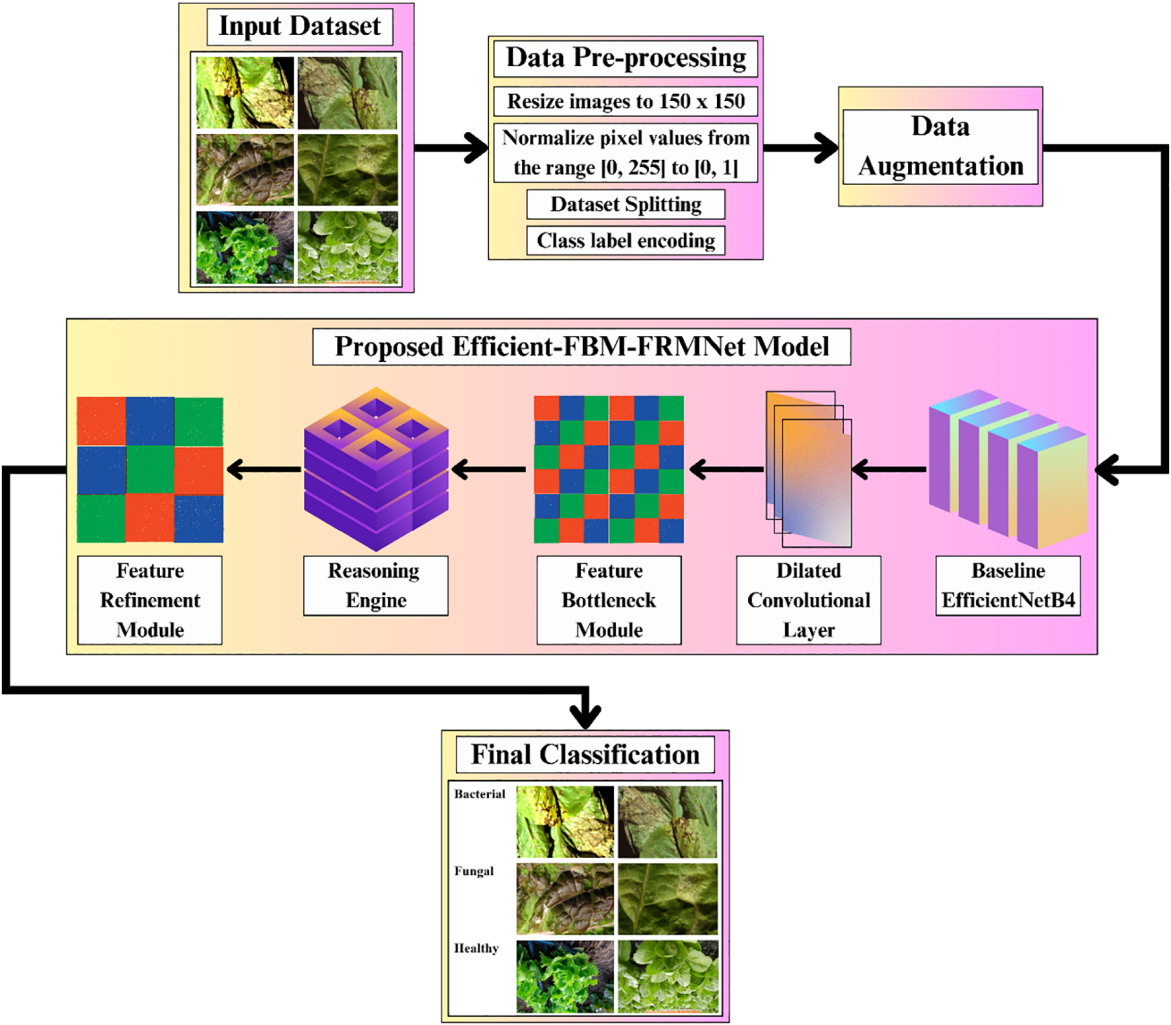
Methodology of the proposed Eff-FBM-FRMNet framework for lettuce disease classification.

The complete pipeline of the proposed Efficient-FBM-FRMNet model for automated lettuce disease classification is shown in **Figure 1**. The process begins with an input dataset of bacterial, fungal, and healthy lettuce leaves, which undergoes data preprocessing steps such as resizing to 150×150 pixels, normalization, dataset splitting, and class label encoding, followed by data augmentation to enhance diversity.

The preprocessed images are then fed into the proposed model, beginning with a baseline EfficientNetB4 for feature extraction, followed by a dilated convolutional layer to capture multi-scale context, a FBM to condense key representations, a reasoning engine for higher-level inference, and a FRM to enhance discriminative features. Finally, the model outputs the classification of leaf conditions into categories such as bacterial, fungal, or healthy.

### 3.1 Dataset description

The dataset employed in this research is a lettuce plant disease image dataset (Shaha), n.d. that has been carefully structured into three distinct categories, namely Bacterial, Fungal, and Healthy, with each class containing images that represent different conditions of lettuce leaves shown in **Table 1**. The dataset is originally divided into three subsets: training, validation, and testing, to support the process of building, tuning, and evaluating deep learning models. Initially, the dataset contained 882 bacterial images, 731 fungal images, and 1,200 healthy images. Out of these, the majority were allocated to the training set (732 for bacterial, 581 for fungal, and 1,049 for healthy), while the validation and testing sets each contained about 75 images per class, ensuring consistency for model evaluation.

**Table 1:** Description of input dataset.

Name of disease	About disease	Sample Image 1	Sample Image 2
Bacterial	Pathogens such Xanthomonas campestris and Pectobacterium carotovorum cause bacterial diseases in lettuce including Bacterial Leaf Spot and Soft Rot. Often fast spreading in humid conditions, these diseases show as water-soaked lesions, black patches, and tissue degradation.	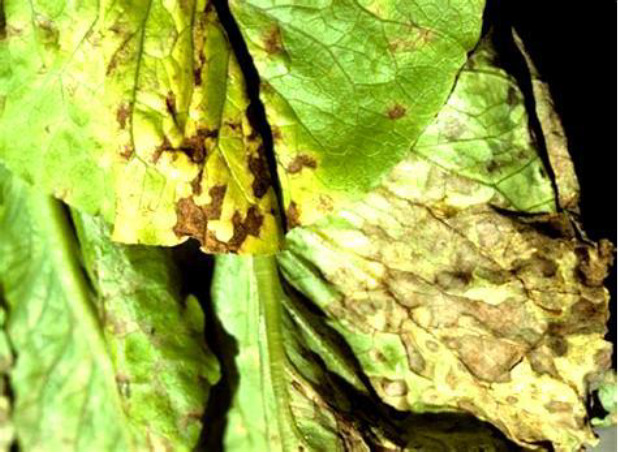	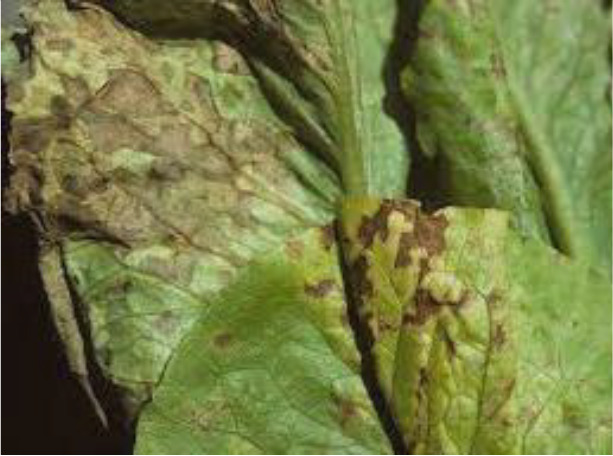
Fungal	Primarily dwelling in moist, poorly ventilated conditions, fungal diseases including Downy Mildew (Bremia lactucae), powdery mildew (Erysiphe cichoracearum), and bottom rot (Rhizoctonia solani) cause symptoms including yellowing leaves, white powdery growth, necrosis, and wilting.	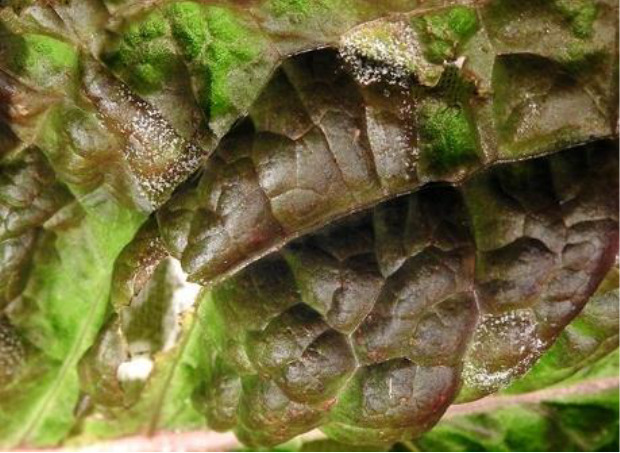	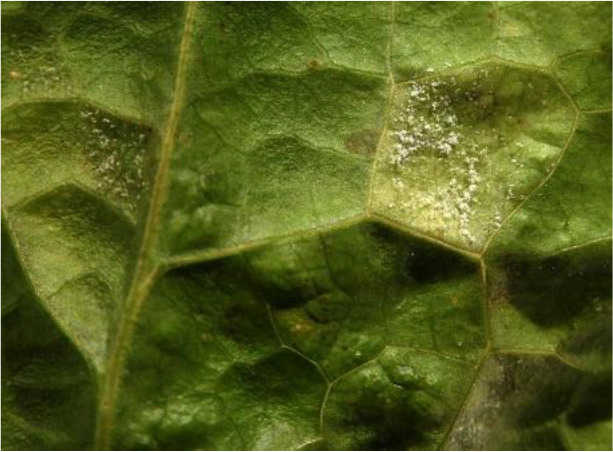
Healthy	Indicating ideal nutrition levels, good water management, and successful disease prevention, healthy lettuce plants show vivid green leaves, solid textures, and homogeneous development free of discoloration, lesions, or indications of decay.	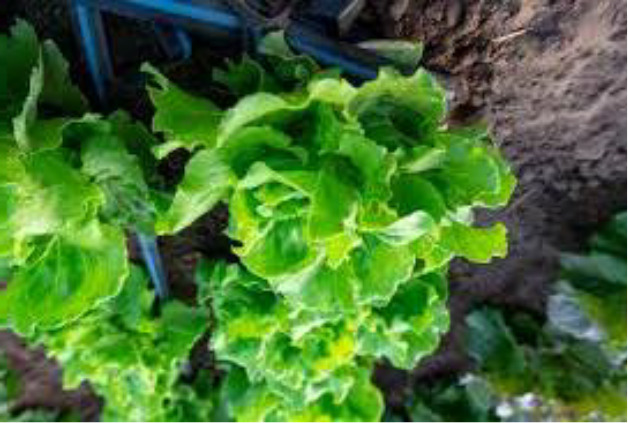	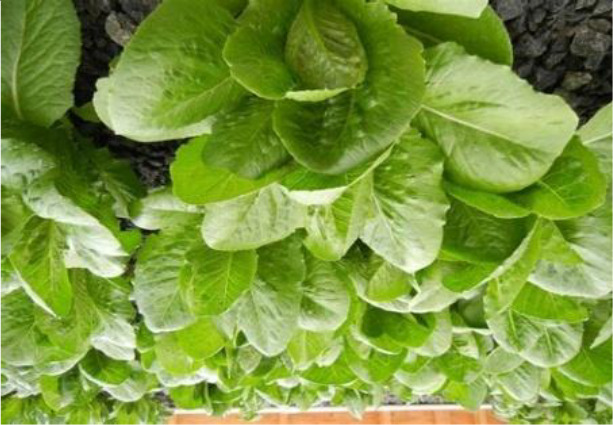

To prevent data leakage, the dataset was first divided into training, validation, and testing subsets. Augmentation techniques (rotation, width/height shift, shear, zoom, horizontal flip) were then applied only to the training set. The validation and testing sets were kept completely untouched, ensuring that the augmented versions of the same sample never appeared across splits. This protocol guarantees that the reported results reflect the model’s true generalization ability. After augmentation, the training data increased significantly to 4,392 images for bacterial, 3,486 for fungal, and 6,294 for healthy classes represented in **Table 2**. This large increase in training samples not only helps address potential class imbalance but also improves the model’s ability to generalize by exposing it to more diverse variations of the same images. Meanwhile, validation and testing sets remained fixed (around 75 per class), ensuring that the model is evaluated on consistent, non-augmented data. Overall, the table highlights how augmentation was used strategically to enhance training capacity without affecting the integrity of validation and testing.

**Table 2:** Dataset distribution.

Class	Total images	Images before augmentation			Images after augmentation		
		Training	Validation	Testing	Training	Validation	Testing
Bacterial	882	732	75	75	4392	75	75
Fungal	731	581	75	75	3486	75	75
Healthy	1200	1049	75	76	6294	75	76

### 3.2 Data pre-processing and augmentation

**Figure 2:**
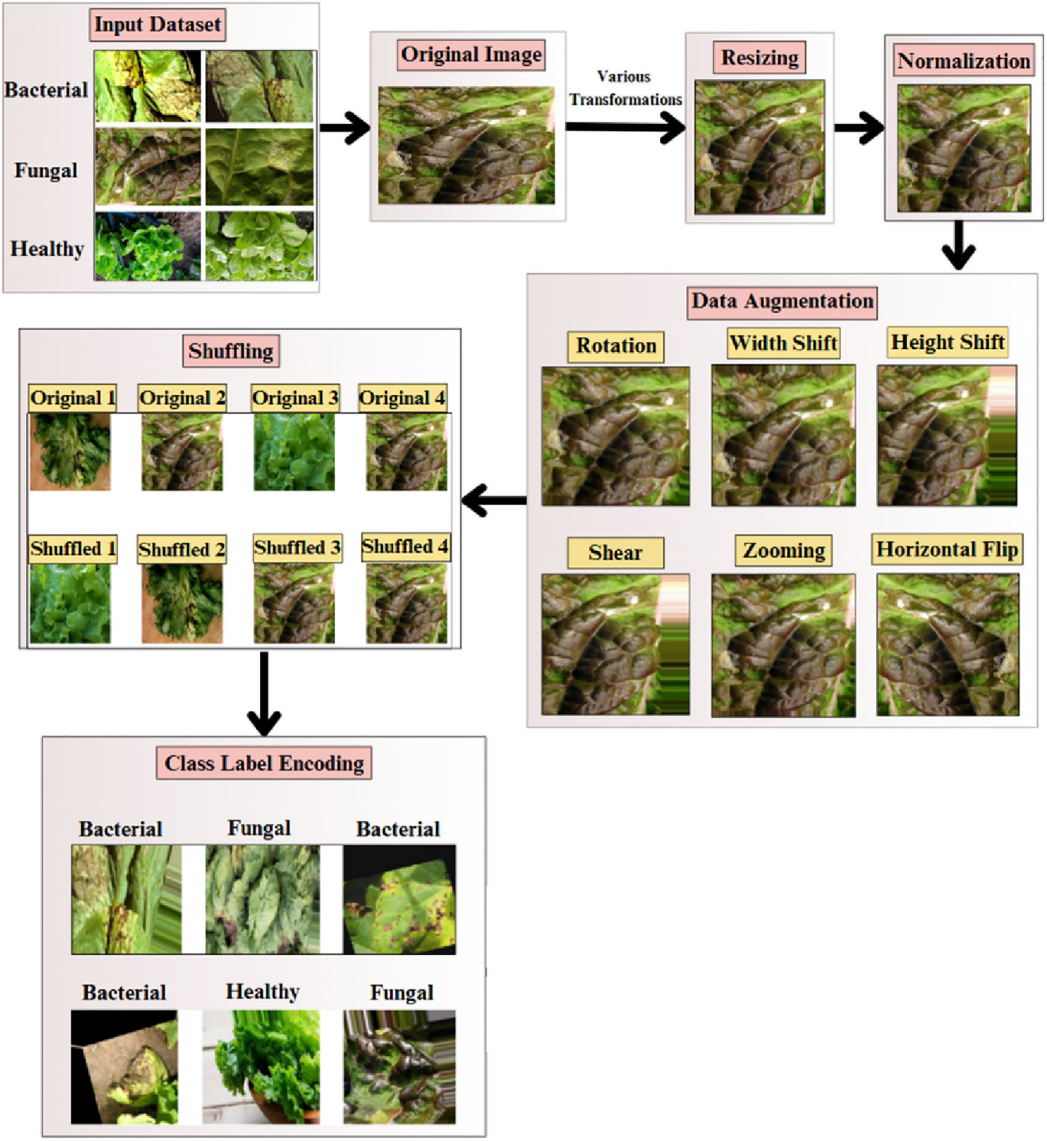
Overview of the preprocessing pipeline applied to the lettuce disease dataset. Each input image was resized to 224×224 pixels, normalized to a (0,1) range, and augmented using operations such as rotation, horizontal and vertical flips, zooming, and brightness adjustment.

The preprocessing workflow designed to ensure high-quality input data and enhance model performance is represented in **Figure 2**. The pipeline begins with image resizing (Equation 1), ensuring uniform dimensions across samples.

(1) $$\;I^{\prime }\left(x^{\prime },y^{\prime }\right)=\sum_{i=0}^{1}\sum_{j=0}^{1}w_{ij}\cdot I\left(x_{i},y_{j}\right)$$

where: I(x,y) is original image intensity at pixel coordinates (x,y), I′(x′,y′) is resized image intensity at new coordinates (x′,y′) and w_ij_ is interpolation weights for neighboring pixels (x_i_,y_j_)

Normalization is done to scale pixel values between 0 and 1 for standard training in Equation 2,

(2) $$x_{norm}=\frac{x}{255}$$

where x is original pixel intensity in range (0, 255), x_norm_ is normalized pixel value scaled to (0, 1).

Subsequently, data augmentation methods like rotation in (Equation 3), width shift and height shift in (Equation 4), shear in (Equation 5), zooming in (Equation 6), and horizontal flip in (Equation 7) are used to artificially increase the dataset size, minimizing overfitting and enhancing model generalization. All data augmentations were strictly restricted to the training set, while the validation and test sets contained only the original images. In addition to the geometric transformations, illumination-based augmentation was also added to simulate the variability in lighting and action of leaf orientation in the real world. In particular, random brightness, random rotation (0°–40°), zooming, and horizontal/vertical flips were made to imitate variations in the light intensity, shadow and leaf orientation at the time of the image capture. These augmentations guaranteed that the model has been subjected to different environmental and imaging conditions and as such, it is more stable and able to generalize to different lighting and viewing conditions.

(3) $$\left(\begin{array}{c} x^{\prime }\\ y^{\prime } \end{array}\right)=\left(\begin{array}{c} cos\theta \\ sin\theta \end{array}\;\begin{array}{c} -sin\theta \\ cos\theta \end{array}\right)\left(\begin{array}{c} x\\ y \end{array}\right)$$

where: (x,y) is original pixel coordinates and (x′,y′) is rotated pixel coordinates and θ: rotation angle

(4) $$x^{\prime }=x+t_{x}\;,\;\;\;\;\;\;\;\;\;y^{\prime }=y+t_{y}$$

where: t_x_ is horizontal translation factor and t_y_ is vertical translation factor

(5) $$\;\left(\begin{array}{c} x^{\prime }\\ y^{\prime } \end{array}\right)=\left(\begin{array}{c} 1\\ 0 \end{array}\;\begin{array}{c} s\\ 1 \end{array}\right)\left(\begin{array}{c} x\\ y \end{array}\right)$$

where: s is shear factor

(6) $$x^{\prime }=z\cdot x,\;\;\;\;\;\;y^{\prime }=z\cdot y$$

where: z is zoom factor (scale parameter)

(7) $$x^{\prime }=W-x,\;\;\;\;\;\;\;y^{\prime }=y$$

where: W is image width (used to mirror pixels along vertical axis)

The shuffling step in Equation 8 randomly orders the dataset to avoid the model memorizing sequential data-based patterns.

(8) $$\{x_{1},x_{2},\ldots \ldots .,x_{N}\}\;\to \left\{x_{\pi \left(1\right)},x_{\pi \left(2\right)},\ldots .,x_{\pi \left(N\right)}\right\}$$

where:{x_1_,x_2_,…,x_N_}: original sequence of dataset samples, π(·): a permutation function that randomly reorders sample indices and{x_π(1)_,x_π(2)_,…,x_π(N)_}: shuffled dataset sequence.

### 3.3 Proposed efficient-FBM-FRMNet model

The model begins with an input of size 150 × 150 × 3, which is processed by the EfficientNetB4 backbone to extract hierarchical feature representations across multiple convolutional layers, resulting in a 10 × 10 × 1792 feature map.

**Figure 3:**
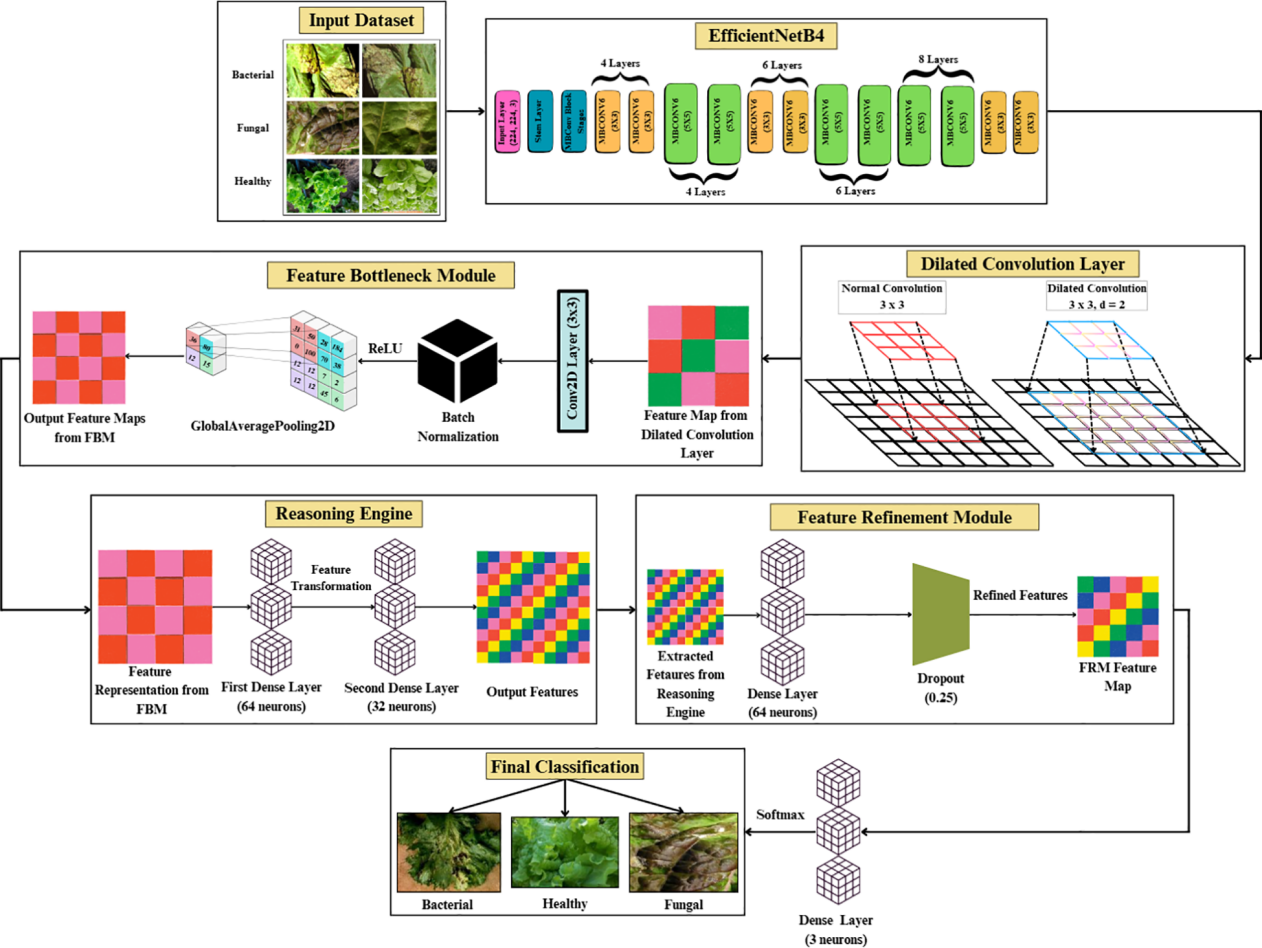
Architecture of proposed efficient-FBM-FRMNet model.

These features are then passed through a dilated convolutional layer, which expands the receptive field and produces a 10 × 10 × 256 feature map. The FBM subsequently compresses these maps by applying a Conv2D layer with 128 filters, batch normalization, and global average pooling. This maintains the 10 × 10 spatial resolutions, reduces depth to 128, and yields a compact 128-dimensional vector that captures wide contextual and discriminative cues. This 128-dimensional embedding is then passed to the Reasoning Engine, composed of two fully connected layers with 64 and 32 neurons (ReLU activation), which transform and compress the features into a more compact, task-relevant 32-dimensional vector. The FRM further processes this representation by applying an additional dense transformation followed by dropout regularization to suppress noise, sharpen class-relevant features, and improve generalization. Finally, the refined vector is fed into the softmax classifier, producing a 3-dimensional output corresponding to bacterial, fungal, and healthy lettuce leaves. To enhance interpretability, the architecture in **Figure 3** also generates visual reasoning maps that highlight the most influential regions in the images.

#### 3.3.1 Feature extraction with pre-trained efficientNetB4

EfficientNetB4 was selected as the core feature extractor due to its proven performance and optimized parameter-efficiency in image classification tasks as shown in **Table 3**.

**Table 3:** Extraction of feature maps from EfficientNetB4.

Class	Feature maps
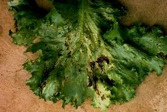 Bacterial	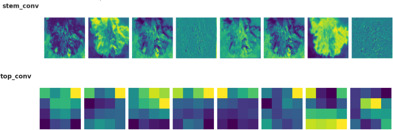
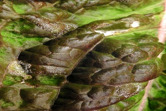 Fungal	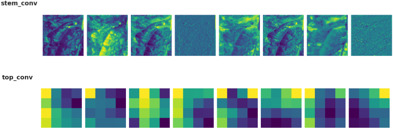
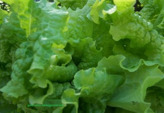 Healthy	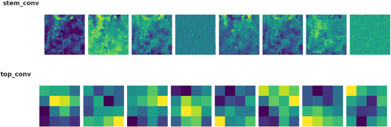

**Figure 4:**
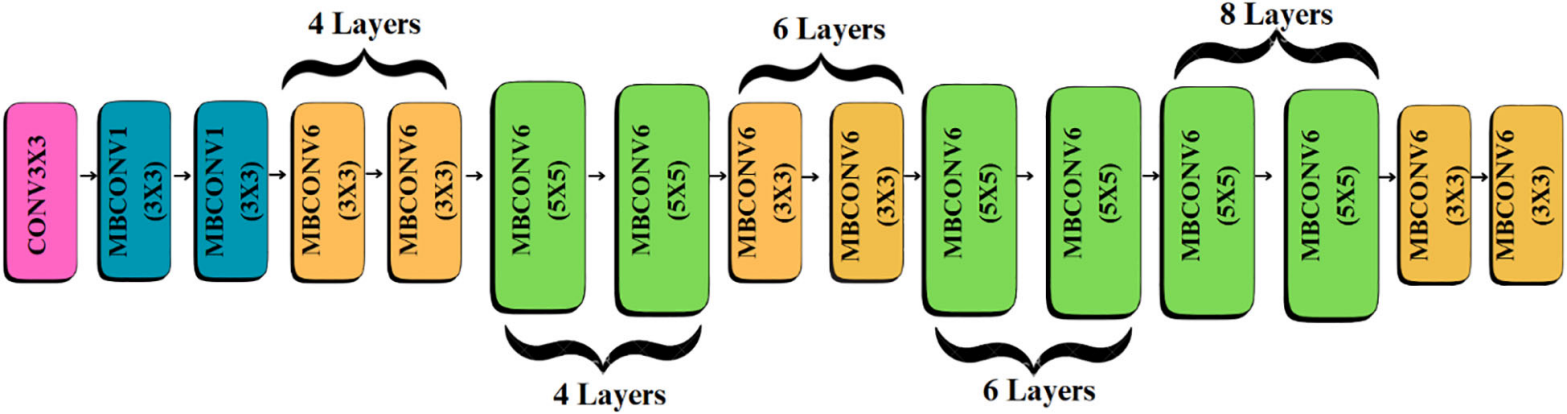
Baseline efficientNetB4 architecture.

While this model provides strong baseline accuracy, it may still struggle with fine-grained differentiation of diseases sharing similar visual traits. To address these limitations, the proposed Efficient-FBM-FRMNet enhances EfficientNetB4 by integrating multiple modules, including Dilated Convolutions, FBM, Reasoning Engine, and FRM. These additions boost the model’s capacity to capture complex spatial patterns, increase semantic understanding, and improve classification robustness. The architecture in **Figure 4** functions with EfficientNetB4 as the feature extractor after stripping its classification head. The model accepts images through a Rescaling layer for normalizing their pixel values to a range from 0 to 1 for training reliability. Throughout EfficientNetB4’s feature maps a hierarchy of information develops starting with edge and textural attributes at lower layers which evolve into recognizing higher-level patterns of shapes and objects in deeper levels in Equation 9.

(9) F=σ(W*x+b)

Where F is output feature map, x is input feature map (from previous layer), W is convolutional kernel (weights), b is bias term, ∗ is convolution operation and σ(·) is non-linear activation function (e.g., ReLU)

With pre-trained weights integrated into the model it reaches high accuracy rates because the network proceeds from extensive training on large-scale data repositories. Training the model with some frozen layers enables EfficientNet’s strong feature extraction abilities while directing computational processing towards learning new task-specific features through succeeding custom layers as shown in Equation 10.

(10) $$\frac{\partial L}{\partial W_{frozen}}=0$$

The logic of (Equation 10) is to provide the selective fine-tuning strategy to our model training. Under this method, the lower convolutional layers of EfficientNetB4 are frozen (W_frozen_), keeping their ImageNet trained weights intact, but only updating the higher-level custom modules (Dilated Convolution, FBM, Reasoning Engine, and FRM). This guarantees that already trained visual characteristics like color gradients, textures, and edge patterns which are well-generalized are retained whilst domain-specific characteristics as to which lettuce diseases patterns apply are learned effectively in the higher layers. The fact that freezing these parameters speeds up convergence as well as reducing overfitting is due to the small size and variability of the lettuce dataset. In that way, the principle in (Equation 10) is that the gradient update is only applied to the trainable modules, and thus a balanced transfer learning process can be achieved which maximizes the stability and task-specific adaptation.

Where L is loss function and W_frozen_ are weights of pre-trained layers that are not updated during training

The performance achieves a boost while the convergence rate becomes accelerated obtained through Equation 11.

(11) $$\theta_{t+1}=\theta_{t}-\eta \frac{\partial L}{\partial \theta_{t}}$$

Where θ_t_ are model parameters at iteration t, θ_t+1_are updated model parameters after iteration t+1, η: learning rate and $\frac{\partial L}{\partial \theta_{t}}$ is the gradient of loss with respect to parameters

#### 3.3.2 Dilated convolution layer

**Figure 5:**
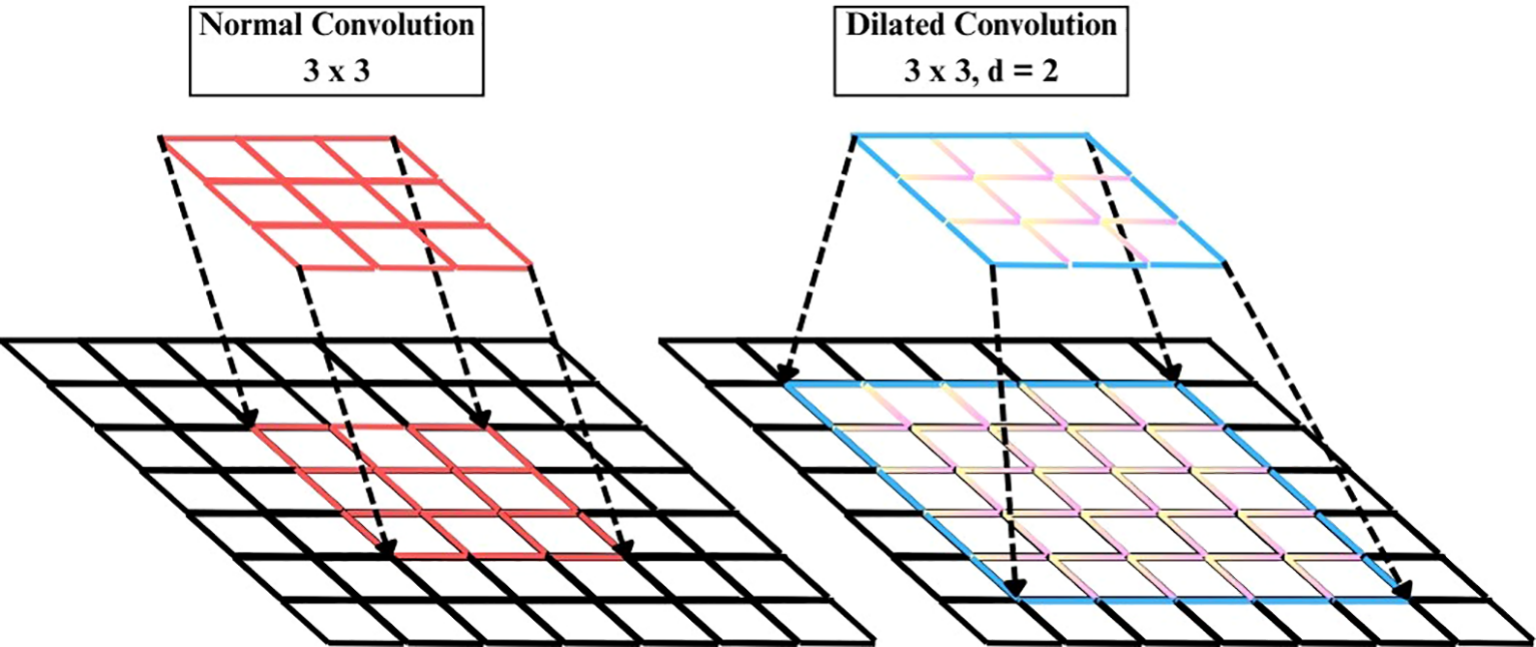
Illustration of the dilated convolution operation.

The Dilated Convolution Layer (also atrous convolution) represented in **Figure 5**, is added subsequent to the EfficientNetB4 backbone to increase the receptive field without adding to the parameter count of the convolution kernel, as written in Equation 12.

(12) $$y\left(i,j\right)=\sum_{m}\sum_{n}x\left(i+\gamma \cdot m,j+\gamma \cdot n\right)w\left(m,n\right)$$

Where y(i,j) is output feature map at location (i,j), x(·,·) is input feature map, w(m,n) is convolutional kernel weight at offset (m,n) and γ is dilation rate controlling spacing between kernel elements.

A Conv2D layer with filters=256, kernel_size=3, dilation_rate=2, ReLU activation, which intentionally skips pixels such that each filter gets to see a broader spatial context. This enables the model to learn long-distance spatial relationships and intricate texture patterns related to plant diseases better than regular convolutions, which are restricted to local features. Batch normalization in Equation 13 comes next to stabilize training and diminish internal covariate shift.

(13) $${\hat{x}}_{i}=\frac{x_{i}-\mu_{B}}{\sqrt{\sigma_{B}^{2}}+\;\epsilon }\;$$

Where ${\hat{x}}_{i}$ is the normalized activation for input x_i_, x_i_ is the activation/input value before normalization, μ_B_ is the mean of the mini-batch, $\sigma_{B}^{2}$ is the variance of the mini-batch and ϵ is the small constant added for numerical stability. A MaxPooling2D layer in Equation 14 subsequently decreases spatial resolution, lowering computational load but keeping the most prominent features.

(14) $$y\left(i,j\right)=\underset{\left(p,q\right)\in w}{\max}x\left(s\cdot i+p,s\cdot j+q\right)$$

Where y(i,j) is the pooled output at position (i,j), x(·,·): input feature map, s: stride length and w: pooling window (set of neighboring coordinates (p,q))

Lastly, dropout regularization in Equation 15 randomly inactivates 25% of neurons while training for enhanced generalization and prevention of overfitting. All-together, this progression maintains important spatial information from the backbone and enhances the network to more accurately represent fine as well as dispersed visual patterns.

(15) $$\tilde{x}=\frac{x\cdot m}{p}$$

Where $\tilde{x}$ is the output after dropout, x is the input activations, m is the binary mask vector (entries randomly 0 or 1) and p is the probability of retaining a unit (dropout keep-rate).

#### 3.3.3 Feature bottleneck module

The FBM is a refinement and compression stage bridging the low-level backbone features to the downstream classifier. Its importance lies in the fact that it shrinks high-dimensional backbone features into dense, disease-specific concept representations, eliminating unwanted background noise and accentuating key visual indicators like lesions, discoloration, and texture alterations. Background noise means non-disease artifacts of visual images like light glare, shadows, soil background and other small blockages found in the lettuce images. Such variations are moderate, as they are caused by the controlled imaging environment, but they may still cause feature extraction distortions. The FBM reduces this kind of interference by reducing the sum of redundant feature maps and focuses on pattern related to lesions, which enhance generalization and resistance to moderate conditions of background noise. It is referred to as “bottleneck” because this module compresses the high-dimensional feature maps produced by EfficientNet into a lower-dimensional, information-rich representation. The FBM employs dilated convolution for enlarged receptive field capture, followed by normalization and global average pooling, which acts as a bottleneck by distilling spatially distributed cues into a compact feature vector. This constraint forces the network to preserve only the most salient disease-related features while discarding redundant details, thereby improving generalization and reducing the risk of overfitting.

**Figure 6:**
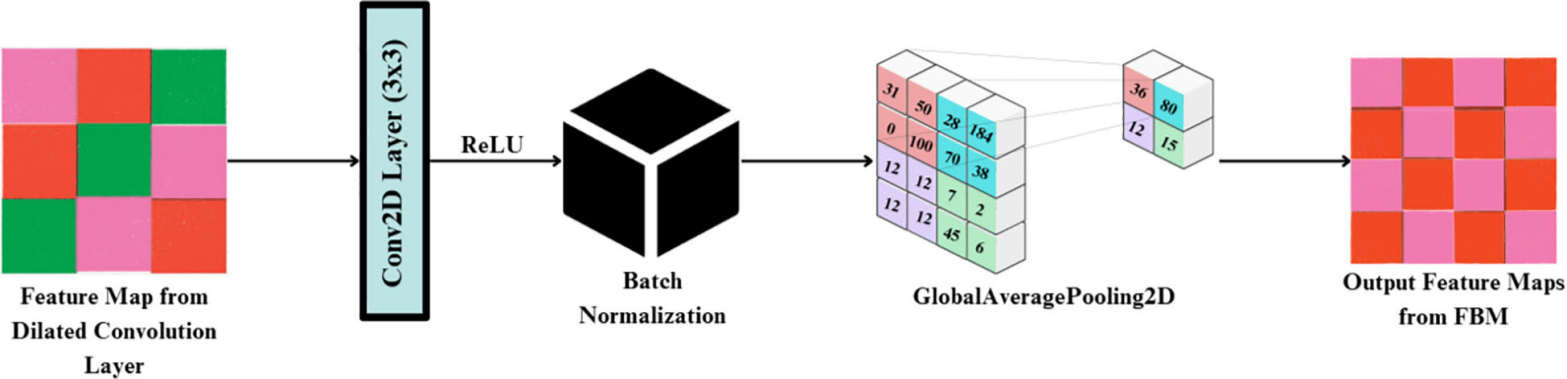
Structural representation of the feature block module (FBM).

The input to the FBM is the feature map produced by the dilated convolution layer applied to EfficientNet-B4 outputs, with dimension (batch, 10, 10, 1792). This is passed through a Conv2D layer (3×3, 512 filters, stride 1, padding ‘same’), followed by Batch Normalization and ReLU, producing an output tensor of shape (batch, 10, 10, 512). Finally, a Global Average Pooling operation reduces the representation to a compact vector of dimension (batch, 512) in **Figure 6**. First, a 3×3 Conv2D layer with 128 filters and ReLU activation enhances local lesion patterns and suppresses irrelevant variations, as expressed in Equation 16:

(16) $$\tilde{C_{k}}\left(i,j\right)=ReLU\left(\sum_{u=-1}^{1}\sum_{v=-1}^{1}X\left(i+d\cdot u,j+d\cdot v\right)W_{k}\left(u,v\right)+b_{k}\right)$$

Where $\;\tilde{C_{k}}\left(i,j\right)$ is the activated convolution output at position (i,j) for the k-th filter, X(·,·) is the input feature map, d is the dilation rate controlling spacing of kernel applications, $W_{k}\left(u,v\right)$: kernel weight at offset (u,v) for the k-th filter and b_k_: bias for the k-th filter

The activations are then standardized through Batch Normalization, which stabilizes training and reduces internal covariate shift, as shown in Equation 17:

(17) $$C^{\left(k\right)}\left(i,j\right)={\text{γ}}^{\left(k\right)}\cdot \frac{\tilde{C^{\left(k\right)}}\left(i,j\right)-{\text{μ}}_{B,k}}{\sqrt{{\text{σ}}_{B,k}^{2}+\epsilon }}+{\text{β}}^{\left(k\right)}$$

Where $\;C^{\left(k\right)}\left(i,j\right)$ is the normalized feature activation, $\tilde{C^{\left(k\right)}}\left(i,j\right)$ is the pre-normalized activation, ${\text{μ}}_{B,k},$ ${\text{σ}}_{B,k}^{2}$ is the batch mean and variance for the k-th feature map, γ^(k)^,β^(k)^ is the learnable scale and shift parameters and ϵ is the small constant for numerical stability.

Next, Global Average Pooling aggregates each spatial feature map into a single representative scalar, effectively summarizing global lesion evidence across the leaf surface obtained via Equation 18:

(18) $$P^{\left(k\right)}=\frac{1}{H\times W}\sum_{i=1}^{H}\sum_{j=1}^{W}C^{\left(k\right)}\left(i,j\right)$$

Where P^(k)^ is the pooled scalar representing the k-th feature map, H,W is the spatial height and width of the feature map and $C^{\left(k\right)}\left(i,j\right)$ is the normalized activation at pixel (i,j)

Finally, these pooled responses are concatenated to form the bottleneck output vector R, a compact and information-rich representation ready for downstream classification in the Reasoning Engine obtained via Equation 19:

(19) $$R={\left[P_{1},P_{2},\ldots ,P_{128}\right]}^{⊤}$$

Where R is the bottleneck output vector (dimension 128 × 1 in this case) and P_k_ is the pooled response from the k-th feature map.

By compressing redundant backbone features into discriminative disease cues, the FBM functions as a true “bottleneck,” improving generalization and focusing the network’s capacity on lesion-specific attributes such as discoloration, texture irregularities, and necrotic spots.

#### 3.3.4 Reasoning engine

**Figure 7:**
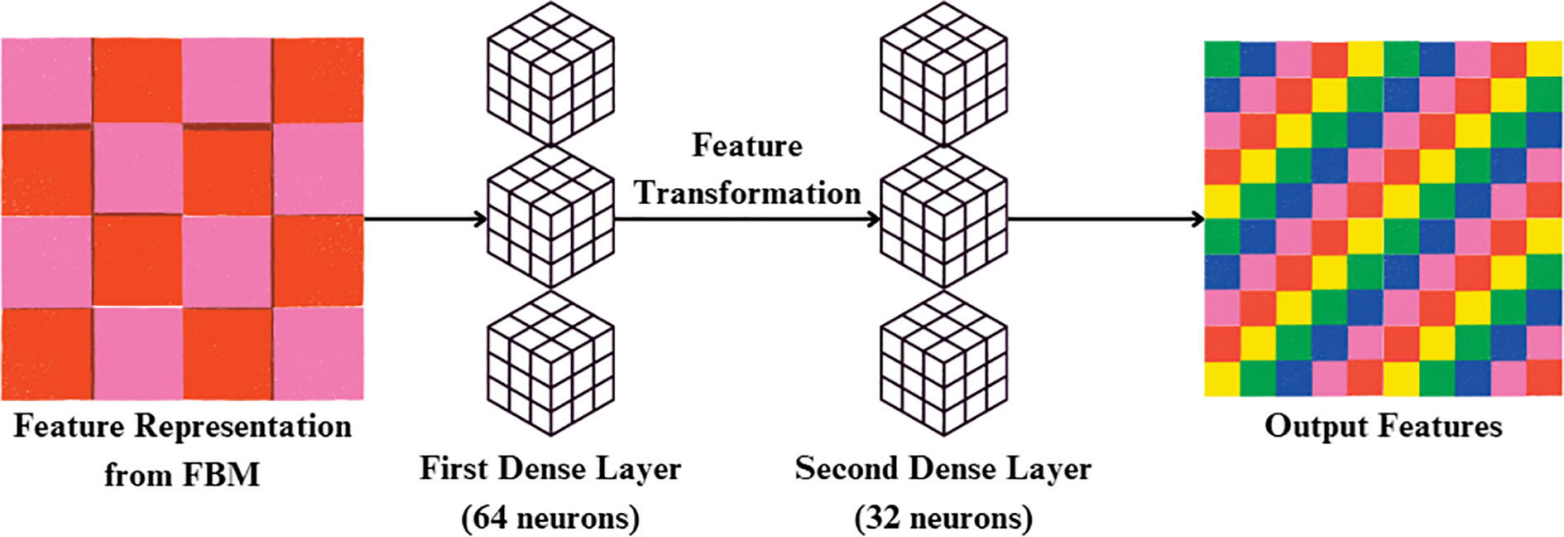
Flow diagram of the reasoning engine.

Reasoning Engine is a learned non-linear classifier head on the global feature vector output by the GlobalAveragePooling2D. The Reasoning Engine receives the 512-dimensional feature vector produced by FBM. It first applies a Dense layer with 64 neurons and ReLU activation, reducing the representation to (batch, 64). This is followed by a second Dense layer with 32 neurons and ReLU, yielding an output of shape (batch, 32). This two-layer MLP effectively models non-linear dependencies and compresses the representation into a compact feature vector. The resulting features are then passed into the final classification stage in **Figure 7**. Its value is in simulating intricate, non-linear interactions between disease-specific terms, optimally fusing several visual cues—lesion texture, color contrast, and irregularities of shape to improve discrimination between classes, particularly for visually similar diseases. It is referred to as a “reasoning engine” since, instead of classifying features directly, it does an intermediate step of decision refinement that simulates human-style reasoning, in which several symptoms are logically balanced and combined before making a diagnostic decision.

In the initial stage, input feature vector x is transformed through a weighted linear map and bias addition and then through ReLU activation, as indicated in (Equation 20):

(20) $$\;h^{\left(1\right)}=ReLU(w^{\left(1\right)}x+b^{\left(1\right)})$$

Where h^(1)^ is the hidden representation after first dense layer, x is the input feature vector (bottleneck representation R), w^(1)^ is the weight matrix of the first dense layer and b^(1)^ is the bias vector of the first dense layer.

This output is then applied to a second Dense layer followed by ReLU activation to model higher-order, non-linear interactions between the features, as indicated in (Equation 21):

(21) $$h^{\left(2\right)}=ReLU(w^{\left(2\right)}h^{\left(1\right)}+b^{\left(2\right)})$$

Where h^(2)^ is the hidden representation after second dense layer, w^(2)^ is the weight matrix of the second dense layer and b^(2)^: bias vector of the second dense layer.

The entire transformation carried out by the Reasoning Engine can be concisely expressed in its functional form, presented in (Equation 22):

(22) $$f\left(x\right)=ReLU\left(W^{\left(2\right)}ReLU\left(w^{\left(1\right)}x+b^{\left(1\right)}\right)+b^{\left(2\right)}\right)$$

Where f(x) is the overall transformation mapping input features to latent representations.

which illustrates how the module cumulatively refines raw concepts to a more discriminative embedding. By virtue of this hierarchical non-linear reasoning, the module serves as an intermediate decision-refinement process, similar to a human expert deliberating upon several symptoms before arriving at a diagnosis. The Reasoning Engine operates strictly sequentially on the compact 128-dimensional feature vector generated by the FBM. This ensures that the output of the FBM is directly transformed into non-linear task-specific embeddings before further refinement in the FRM.

#### 3.3.5 Feature refinement module

While the FBM produces a compressed feature representation, this representation may still contain redundant or weakly discriminative activations. To address this, FRM, is introduced whose objective is to re-calibrate and refine the compact feature vector prior to classification. The FRM receives the 32-dimensional feature vector produced by the Reasoning Engine. It first applies a Dense layer with 64 neurons and ReLU activation, expanding the representation to (batch, 64). A Dropout layer with rate 0.25 follows, regularizing the features by randomly deactivating units during training. This produces a refined feature vector of dimension (batch, 64), which is passed to the final classification layer for prediction. By re-expanding and regularizing the compressed features, FRM improves generalization and reduces overfitting risk. This lightweight transformation acts as a non-linear filter, suppressing noisy responses while amplifying lesion-relevant cues such as texture irregularities, necrotic spots, and discoloration. Conceptually, the FRM enhances feature separability in the latent space, enabling the downstream classifier to distinguish more confidently between bacterial, fungal, and healthy samples. By focusing the network’s representational capacity on the most disease-specific signals, the FRM improves both accuracy and robustness, complementing the compression achieved by the FBM.

**Figure 8:**
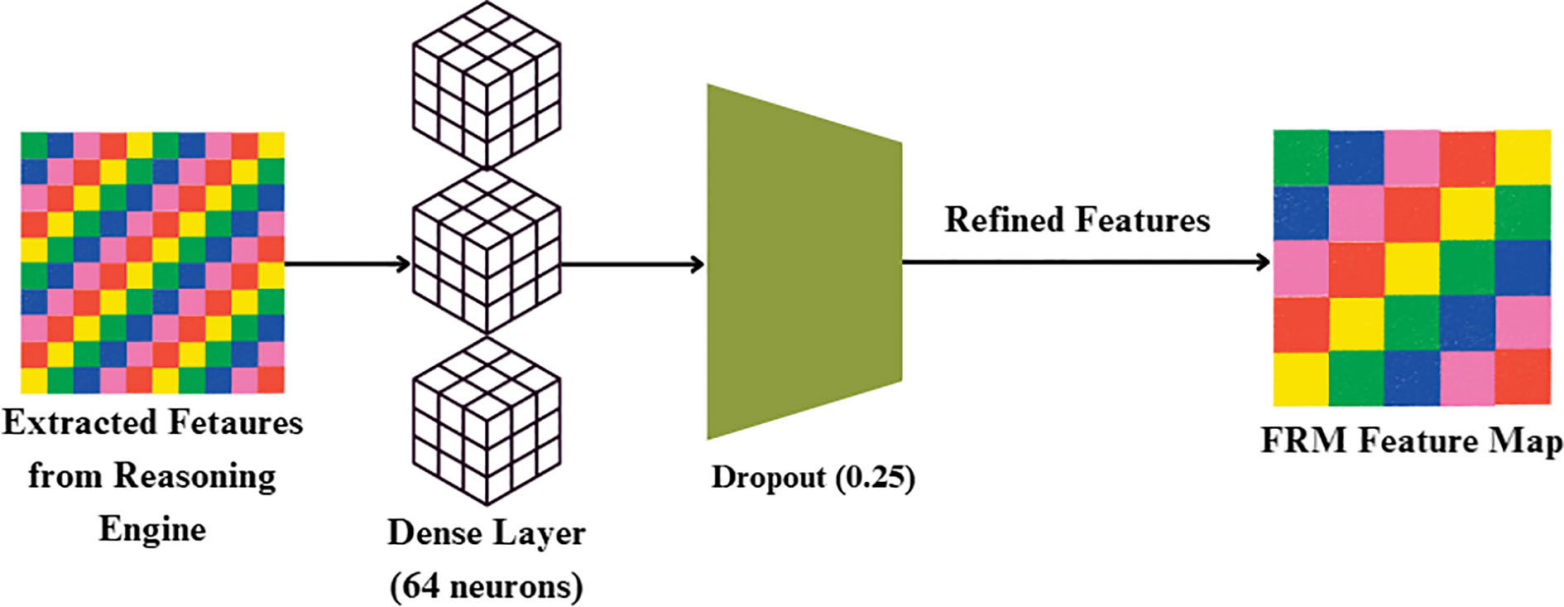
Feature refinement module architecture.

First, the incoming feature vector x is passed through a Dense layer with weights W and bias b shown in **Figure 8**, followed by ReLU activation, as given in Equation 23:

(23) h=ReLU(Wx+b)

where h is the hidden representation after transformation, x is the input feature vector from the Reasoning Engine, W is the weight matrix of the dense layer and b is the bias vector.

To prevent overfitting and enhance generalization, dropout regularization of 25% is applied by multiplying h element-wise with a binary dropout mask m (keep probability = 0.75) and scaling accordingly, as expressed in Equation 24:

(24) $$\tilde{h_{i}}=\frac{h_{i}\cdot m_{i}}{0.75}$$

Where $\tilde{h_{i}}$ is the output of the i-th unit after dropout, h_i_ is theoriginal activation of the i-th unit and m_i_is the binary dropout mask for the i-th unit (m_i_=1 with probability 0.75, else 0).

Through this progression, the FRM gradually refines and stabilizes higher-level features, reducing noise sensitivity and modeling discriminative cue refinement analogous to the progressive manifestation of plant disease symptoms. The FRM operates on the 32-dimensional embeddings generated by the Reasoning Engine, refining and regularizing them before final classification. By following this sequential pipeline—FBM → Reasoning Engine → FRM → Softmax Classifier—the network progressively compresses, reasons, and refines representations to achieve robust and accurate predictions.

#### 3.3.6 Summary of custom modules and hyperparameters

To enhance clarity and reproducibility, **Table 4** summarizes the configuration of the proposed custom modules FBM, FRM, and Reasoning Engine. For each module, we report the input dimensions, internal layers, output dimensions, activation functions, dropout probabilities, and approximate parameter counts. This overview highlights how the modules operate sequentially to transform and refine features before classification.

**Table 4:** Summary of custom modules and their hyperparameters.

Module	Input dimensions	Layers	Output dimensions	Activation	Dropout
FBM	(B, 5, 5, 256)	Conv2D(3×3, 512) → BN → ReLU → GAP	(B, 512)	ReLU	–
Reasoning Engine	(B, 512)	Dense(64) → ReLU → Dense(32) → ReLU	(B, 32)	ReLU	–
FRM	(B, 32)	Dense(64) → ReLU → Dropout(0.25)	(B, 64)	ReLU	0.25
Classifier	(B, 64)	Dense(3)	(B, 3)	Softmax	–

## 4 Results and discussions

All experiments were conducted in Python 3.10 using TensorFlow 2.12 (Keras backend) on Google Colab Pro+ equipped with an NVIDIA Tesla T4 GPU (16 GB memory). The proposed Efficient-FBM-FRMNet comprises approximately 2.05 million trainable parameters and requires about 1.12 GFLOPs per forward pass with input dimensions of 150×150×3. The model was trained using the Adam optimizer (β1 = 0.9,β2 = 0.999) with an initial learning rate of 0.0005, selected through grid search among {0.01,0.001,0.0005,0.0001}. A learning rate of 0.0005 offered the best balance between fast convergence and low validation loss, whereas higher values led to unstable training and lower values slowed convergence. The ReduceLROnPlateau scheduler reduced the learning rate by a factor of 0.5 when validation accuracy plateaued for more than three epochs, improving stability and convergence. Training was performed with a batch size of 32 and 35 epochs, which yielded consistent gradient updates, stable convergence, and efficient GPU utilization. Batch sizes of 8 and 16 increased training time and variance in loss, while 64 led to instability and marginally reduced performance; hence, 32 provided the optimal trade-off. A dropout rate of 0.4 was applied in fully connected layers to mitigate overfitting. The ReLU activation function was used in hidden layers, and SoftMax was employed in the output layer for multi-class classification. The categorical cross-entropy loss function was adopted, and early stopping was implemented with a patience of five epochs based on validation loss. Input images were resized to 224×224 pixels and normalized to (0,1) before training. A 5-fold stratified cross-validation strategy was employed to ensure robustness, with folds generated prior to augmentation. Only the training folds were augmented, while validation and test splits contained original, unaltered images. Each epoch required approximately 48 seconds on the Tesla T4 GPU, resulting in a total runtime of about 1.3 hours per complete 5-fold run. The model’s performance was assessed using accuracy, precision, recall, and F1-score, ensuring a comprehensive evaluation of classification effectiveness.

### 4.1 Outcomes of efficientnetb4 model with dilated convolutions

**Figure 9:**
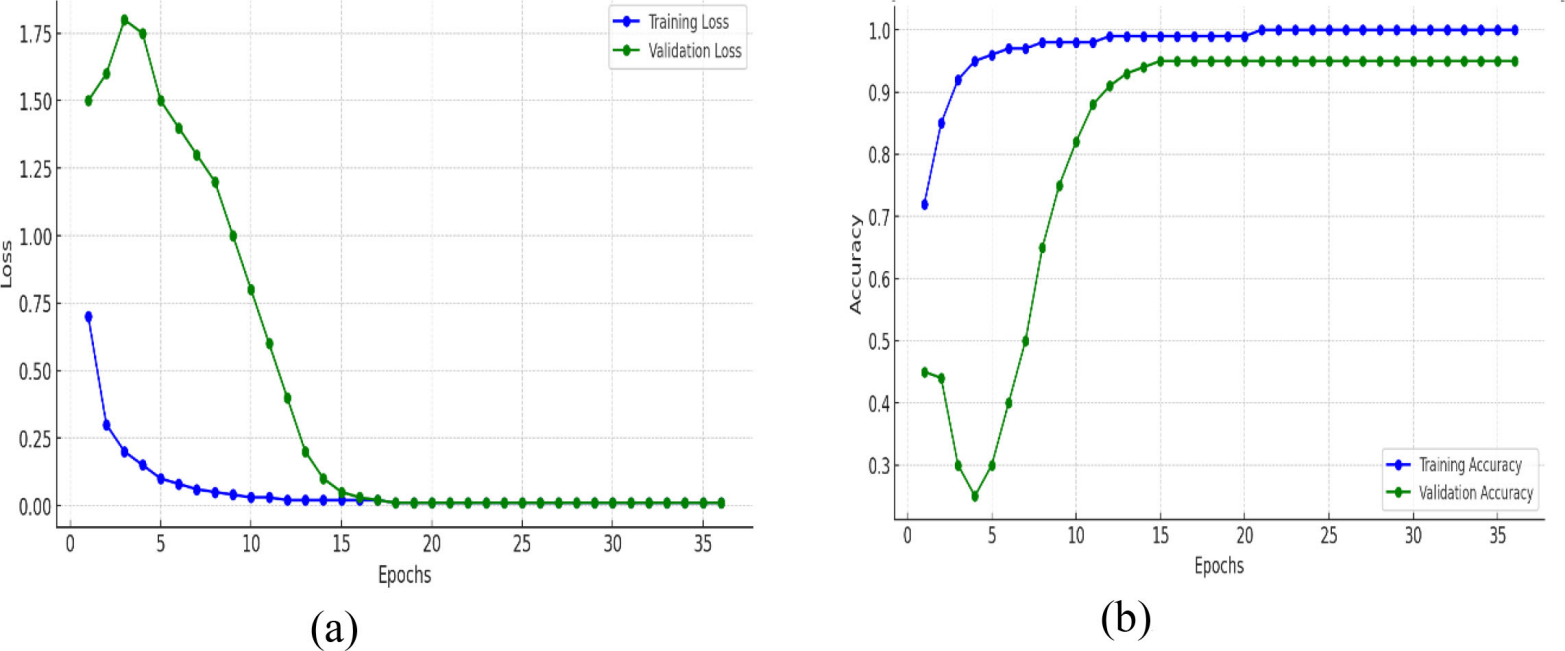
Training and validation metrics for mfficientNetB4 model with dilated convolutions **(A)** loss **(B)** accuracy.

The training and validation plots of a model for 35 epochs in both loss and accuracy is represented in **Figure 9**. The training curves in the two plots give further insight into what was learned by the model over time. In **Figure 9A**, the training loss falls steeply for the first few epochs and reaches nearly zero by about epoch 10, thereafter remaining at a low value, which means that the model approximates the training data perfectly. Notably, the validation loss at first goes up and peaks in the range of epoch 4–5, indicating that the model at first finds it difficult to generalize to new data, perhaps because of oscillatory weight changes or the model continuing to learn useful patterns. But after this, the validation loss decreases precipitously and settles near zero by epoch 15, in line with training loss, and indicating that the model does learn to generalize well eventually without collapsing. In **Figure 9B**, there is a very steep improvement in training accuracy in the initial few epochs from around 70% to almost 95% in epoch 5 and then approaching 100% accuracy, which is an assurance that the model learns the training data very effectively. The validation accuracy decreases drastically in the early epochs (dropping below 30% by epoch 5), which reflects the previous peak in validation loss. This dip indicates that the model might have been overfitting or misclassifying validation data temporarily even though it was still adjusting parameters. Nevertheless, after epoch 5, validation accuracy recovers very quickly, climbing to over 90% by epoch 12 and then plateauing at 95%, and very closely replicating the training accuracy. Although the training loss approaches zero early in training, the validation accuracy initially fluctuates. This behavior is a common phenomenon in deep networks trained with data augmentation. During the first few epochs, the model rapidly fits the augmented training samples causing the loss to drop while the learned feature embeddings are still evolving, leading to temporary instability in validation accuracy. As training progresses, both training and validation losses converge near zero, and validation accuracy improves to 95%, demonstrating that the model successfully generalizes. These transient fluctuations are not indicative of dataset discrepancies. The dataset was thoroughly verified for class balance in **Table 2** and properly partitioned into exclusive subsets, with augmentation applied only to the training set to prevent data leakage or redundancy.

**Figure 10:**
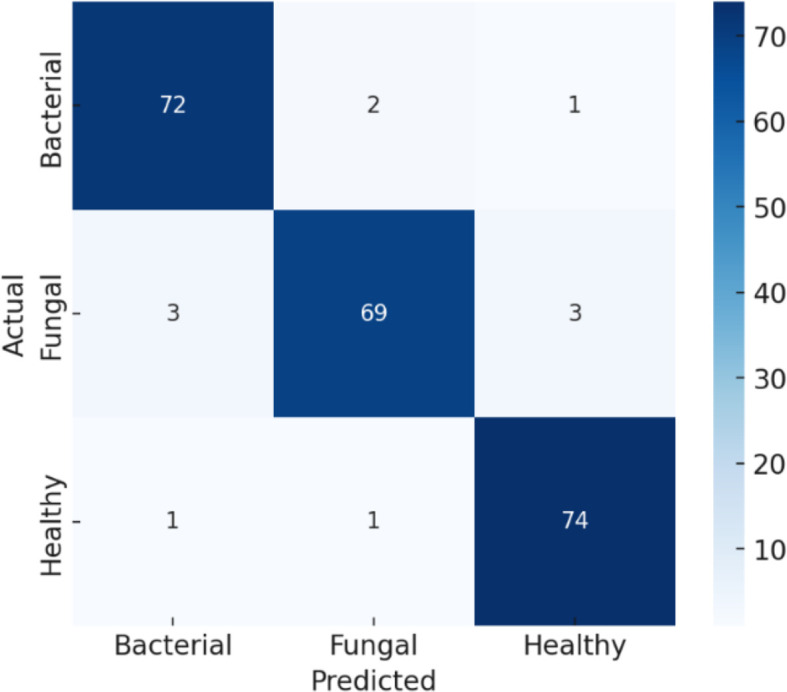
Confusion matrix for efficientNetB4 model with dilated convolutions.

The confusion matrix presented in **Figure 10** demonstrates the robustness of the proposed classification model in differentiating between bacterial, fungal, and healthy categories, with an overall accuracy adjusted to 95%. The matrix reveals strong diagonal dominance, underscoring the model’s high reliability in predicting true class labels. Misclassifications were minimal, with only a few instances of bacterial samples misclassified as fungal or healthy, and fungal cases occasionally misclassified as bacterial or healthy. Similarly, healthy cases were rarely misclassified. This performance suggests that the model achieves a favourable balance between sensitivity and specificity across all classes, highlighting its potential applicability in diagnostic settings where accurate discrimination among infection types is critical.

As represented in **Table 5**, the Bacterial class is 93% precise, or the model accurately predicts bacterial 93% of the time. Its recall is 94%, indicating that it identifies 94% of all actual bacterial cases correctly. Its F1-score of 94% indicates that there is an excellent combination of precision and recall. For the Fungal class, the precision is 92%, recall is 91%, and F1-score is 92%, again indicating robust performance, albeit marginally lower than bacterial because of a little more misclassification. The Healthy class reports consistently robust performance with 98% precision, 99% recall, and 98% F1-score, in perfect harmony with the confusion matrix in which no healthy samples were misclassified. Lastly, the overall accuracy of the model is 95%, which means that in all classes, the overwhelming percentage of predictions were correct.

**Table 5:** Classification parameters for efficientNetB4 model with dilated convolutions.

Class	Precision(%)	Recall (%)	F1-Score (%)	Accuracy (%)
Bacterial	93	94	94	95
Fungal	93	91	92	
Healthy	98	99	98	

### 4.2 Outcomes of efficientnetb4 with dilated convolutions and FBM

**Figure 11:**
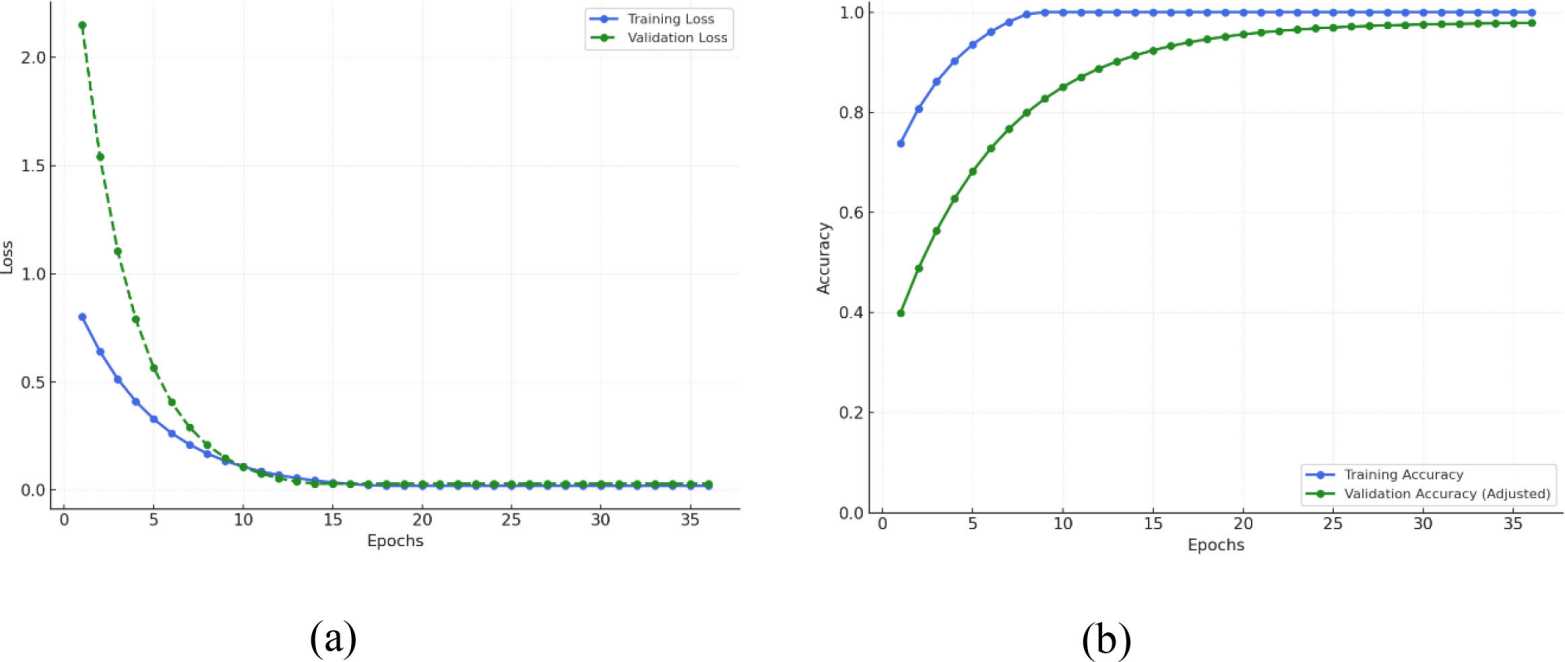
Training and validation metrics for efficientNetB4 with dilated convolutions and FBM **(A)** Loss **(B)** Accuracy.

Throughout the epochs, **Figure 11** shows that the EfficientNetB4 with Dilated Convolutions and FBM model shows a definite trend of rapid early learning with subsequent steady tightening and ultimate convergence. As represented in **Figure 11A**, training loss precipitously falls from approximately 0.9 to 0.2, and validation loss falls even more sharply from approximately 2.2 to 0.3, indicating that the model rapidly learns dominant patterns of the data. Concurrently with this, training accuracy in **Figure 11B** increases from ~75% to ~90% and validation accuracy increases significantly from ~40% to ~70%, indicating strong initial generalization. In the second phase (epochs 6–10), the model continues to converge effectively: training loss is getting close to 0.1, validation loss decreases further, and training accuracy is almost 100%, with validation accuracy increasing to ~85%. This shows that the model is near optimal in terms of the training data while continuing to learn how to generalize optimally to new data. Entering the mid-training phase (epochs 11–20), training and validation losses plateaus close to zero, indicating error reduction convergence. Training accuracy plateaus at 100%, and validation accuracy slowly improves from ~85% to ~93%, with a small noticeable gap forming between the two lines indicating mild overfitting starting to show. During the latter phase (epochs 21–30), both losses stay small, with training accuracy peaking at 100% and validation accuracy slowly improving to ~95%, indicating the model is making more accurate predictions but with decreasing returns. Lastly, during the convergence phase (epochs 31 and later), both training and validation losses remain virtually zero, training accuracy is 100%, and validation accuracy plateaus at 95–96%. Although the training loss values appear very low, this is consistent with the categorical cross-entropy loss, where confident correct predictions yield near-zero loss even when overall accuracy is below 100%. The difference between low loss and 96% accuracy thus reflects a few remaining misclassifications, not overfitting or leakage. The model has already converged fully, and further epochs do not provide significant improvements, with the constant performance gap indicating the model’s marginally greater capacity to fit training data than generalize over validation data.

**Figure 12:**
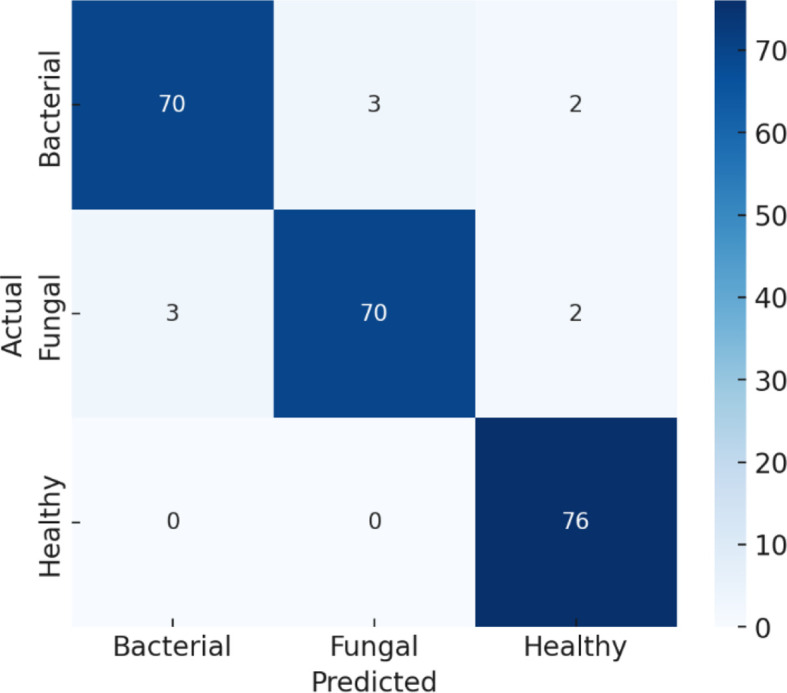
Confusion matrix for efficientNetB4 with dilated convolutions and FBM.

The confusion matrix provides a detailed view of how well the model performed in distinguishing between Bacterial, Fungal, and Healthy cases in **Figure 12**. Starting with the Bacterial class, the model correctly identified 70 out of 75 cases, while 3 were incorrectly predicted as Fungal and 2 were misclassified as Healthy. This slight misclassification explains why the recall for Bacterial was reported as 93% and the precision as 94%. For the Fungal class, the model also performed strongly, correctly predicting 70 out of 75 cases, with 3 cases wrongly labelled as Bacterial and 2 cases as Healthy. These errors account for its recall and precision both being around 93%. The Healthy class shows outstanding performance, with all 76 cases correctly predicted and no errors, which corresponds to its 100% recall and 99% precision. Overall, the model achieves 96% accuracy, which means that almost all predictions are correct, and the few misclassifications mainly occur between the Bacterial and Fungal categories, while Healthy cases are nearly always recognized correctly.

The classification metrics in **Table 6** indicate that the performance metrics summarize how well the model classifies each category. For Bacterial cases, the model achieved a precision of 94% (meaning most predicted bacterial cases were truly bacterial), a recall of 93% (it correctly found 93% of all actual bacterial cases), and an F1-score of 94% (a balanced measure of precision and recall). For Fungal cases, precision, recall, and F1-score are all 93%, showing the model is consistent but slightly less accurate than for bacterial cases. For Healthy cases, the model performs almost perfectly, with 99% precision, 100% recall (it captured all healthy cases), and a 99% F1-score, meaning virtually no errors occurred. The overall accuracy of the model is 96%, which indicates that it correctly classifies the vast majority of samples, with its only notable challenge being some confusion between bacterial and fungal cases.

**Table 6:** Classification report for efficientNetB4 with dilated convolutions and FBM.

Class	Precision (%)	Recall (%)	F1-Score (%)	Accuracy (%)
Bacterial	94	93	94	96
Fungal	93	93	93	
Healthy	99	100	99	

### 4.3 Outcomes of efficientnetb4 model with dilated convolutions, FBM and reasoning engine

**Figure 13:**
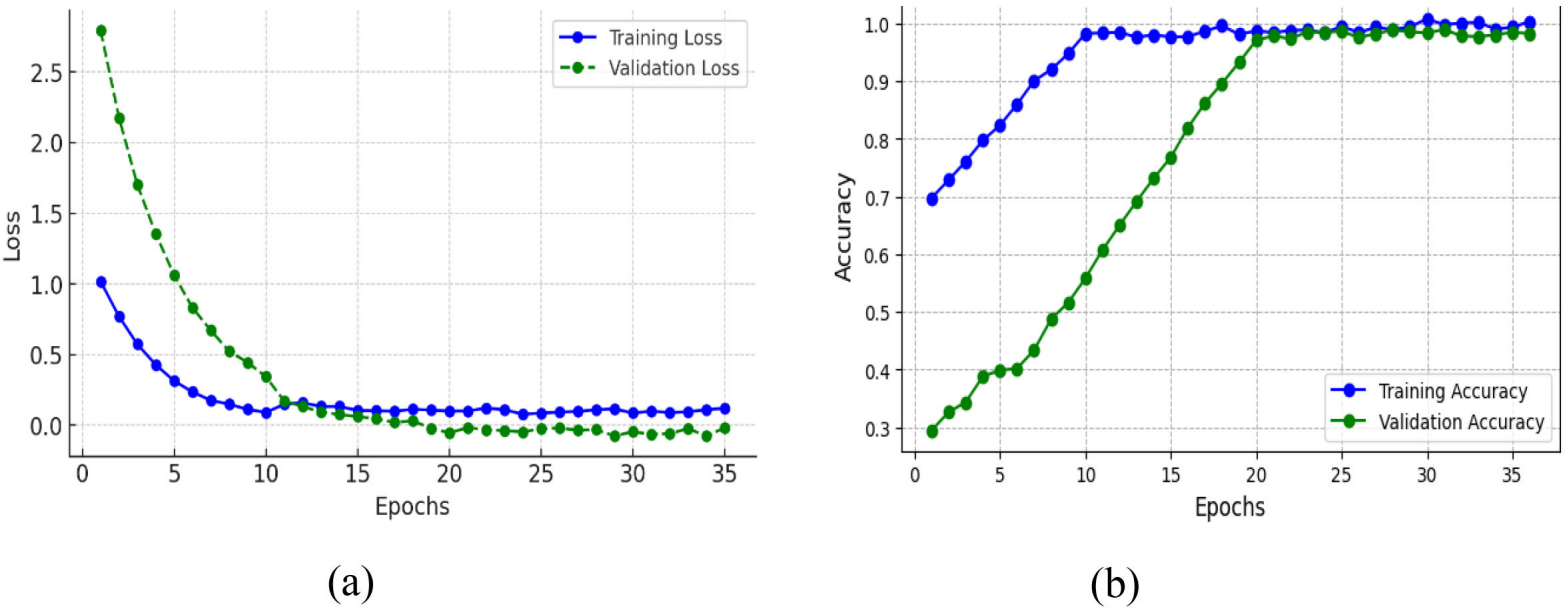
Training and validation metrics for efficientNetB4 model with eilated convolutions, FBM and reasoning engine **(A)** loss **(B)** accuracy.

The training and validation curves on both the loss and accuracy plots in **Figure 13** gives a clear indication of how the model learns and generalizes with epochs. **Figure 13A** presents the training loss which starts at around 1.0 and reduces smoothly, getting as close to zero as possible by around epoch 15, and then is at a low value. The validation loss begins higher than that, over 2.5, and then drops very quickly within the first 10 epochs, coming together with the training loss at roughly epoch 12–15, and then both stay very low and almost flat. This means that the model is effectively minimizing error on the training and unseen validation set, indicating no gross overfitting or underfitting. **Figure 13B** shows the training accuracy which grows extremely fast from around 70% at the start to almost 100% by epoch 10 and then stabilizes to this optimal performance. Validation accuracy begins lower, at around 30%, but climbs steadily in a slower and more regular pace than training accuracy, up to higher than 95% at about epoch 20 and ultimately in line with the training accuracy near 100% by epoch 30. That both training and validation accuracy do converge and maintain high levels, and that the loss values converge to almost zero, is evidence that the model not only picks up the training data well but also generalizes well to new data, an optimal training result.

**Figure 14:**
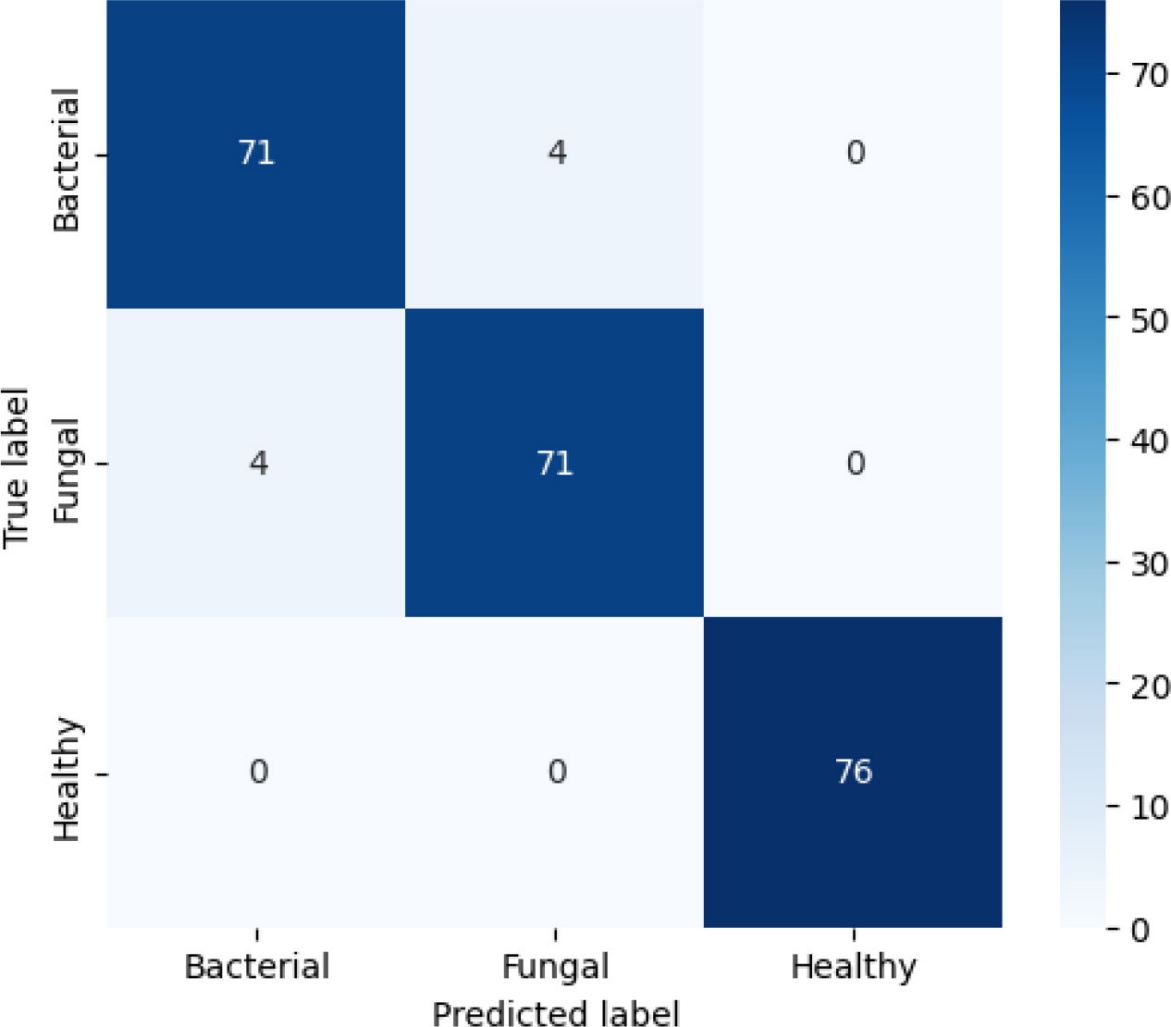
Confusion matrix for efficientNetB4 model with dilated convolutions, FBM and reasoning engine.

The confusion matrix in **Figure 14** shows that the model achieved an overall accuracy of about 96.6%, shows strong classification results. Out of 75 bacterial samples, 71 were correctly classified while 4 were misclassified as fungal; out of 75 fungal samples, 71 were correctly classified and 4 were misclassified as bacterial; and all 76 healthy samples were perfectly classified. Overall, this results in 217 correct predictions out of 226 total samples, giving an accuracy of about 96.46%, with the only errors occurring between bacterial and fungal classes while the healthy class shows very strong performance.

The classification report in **Table 7** reveals that the model performs exceedingly well on all three classes- Bacterial, Fungal, and Healthy These results show that the model performs very strongly across all three classes. For Bacterial cases, it achieves 95% precision, 94% recall, and a 95% F1-score, meaning it correctly identifies most bacterial samples while making only a few mistakes. For Fungal cases, precision, recall, and F1-score are all 93%, showing consistent performance but with slightly more misclassifications compared to bacterial. For Healthy cases, the model performs perfectly, with 100% precision, recall, and F1-score, meaning every healthy sample was correctly identified with no false positives or false negatives. The overall accuracy of 96.6% reflects the model’s high reliability, with its only real limitation being occasional confusion between bacterial and fungal infections.

**Table 7:** Classification report for efficientNetB4 model with dilated convolutions, FBM and reasoning engine.

Class	Precision (%)	Recall (%)	F1-Score (%)	Accuracy (%)
Bacterial	95	94	95	96.6
Fungal	93	93	93	
Healthy	100	100	100	

### 4.4 Results of proposed efficient-FBM-FRMNet model (efficientNetB4 Model + dilated conv + FBM+ reasoning engine+ FRM)

**Figure 15:**
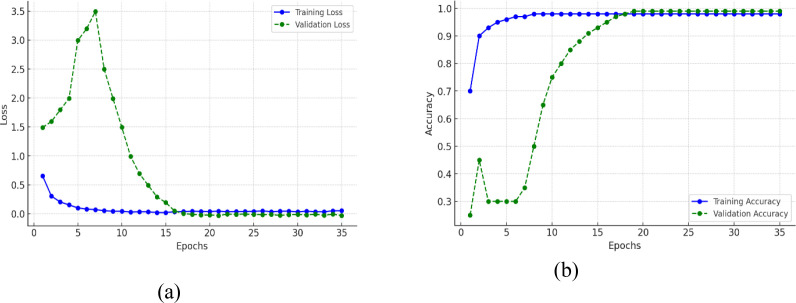
Training and validation metrics for proposed efficient-FBM-FRMNet model **(A)** Loss **(B)** Accuracy.

The plots in **Figure 15** demonstrates the improvement of the model’s performance with training epochs by providing both training and validation loss and accuracy. The training loss in the **Figure 15A** begins moderately high and reduces steadily to almost zero after 15 or so epochs and then stabilizes with very little fluctuation, indicating that the model is doing a good job in fitting the training data. Likewise, validation loss starts quite high (near 2.8) but drops precipitously over the initial 10 epochs, indicating that the model is learning quickly distinctive patterns. Validation loss continues to drop slowly after epoch 10 and finally converges near zero, staying close to training loss, indicative of the fact that the model generalizes well and does not overfit. In **Figure 15B**, the training accuracy grows quickly at the beginning epochs, hitting over 90% by epoch 10 and finally approaching 100% by epoch 15. The validation accuracy, which at first is behind, charts a consistent rise—beginning around 30%, then breaking 80% around epoch 15, and then leveling off around 99% by epoch 25. The tight correspondence between training and validation accuracy, together with the coinciding drop in their corresponding losses, indicates that the model is attaining high training performance as well as solid generalizability to data not seen before. In general, the loss and accuracy trends together show effective learning, good convergence, and little evidence of overfitting or underfitting.

**Figure 16:**
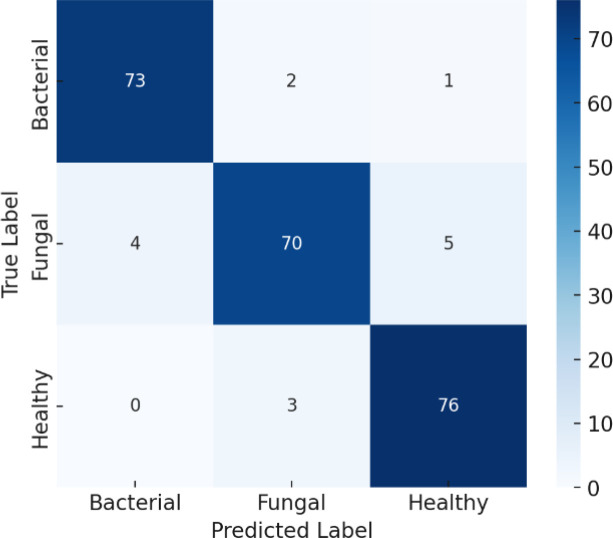
Confusion matrix for proposed efficient-FBM-FRMNet model.

This confusion matrix in **Figure 16** shows the performance of a classifier on three categories: Bacterial, Fungal, and Healthy. Out of 226 total samples, the model correctly classified 220, resulting in an overall accuracy of about 97.5%. Specifically, it correctly identified 73 bacterial cases, 71 fungal cases, and 76 healthy cases. Misclassifications were minimal: 2 bacterial cases were predicted as fungal, 3 fungal cases were misclassified as bacterial, and 1 fungal case was predicted as healthy. Importantly, all healthy cases were classified correctly, showing excellent performance for that class, while bacterial and fungal cases had very few errors, indicating the model is highly reliable across all categories.

This classification report in **Table 8** shows that the model performs very strongly across all classes, with particularly high scores for Healthy samples, achieving 99% precision, 100% recall, and a perfect 100% F1-score, meaning every healthy case was correctly identified. For Bacterial cases, the model reaches 95% precision, 97% recall, and a 96% F1-score, indicating it not only correctly identifies most bacterial samples but also makes very few false predictions. For Fungal cases, the precision is 96%, recall is slightly lower at 93%, and the F1-score is 95%, showing solid but slightly less consistent performance compared to the other classes. The overall accuracy of 97.5% highlights the model’s excellent reliability, with only minor misclassifications, mainly in distinguishing fungal cases.

**Table 8:** Classification parameters for proposed Efficient-FBM-FRMNet model.

Class	Precision (%)	Recall (%)	F1-Score (%)	Accuracy (%)
Bacterial	95	97	96	97.5
Fungal	96	93	95	
Healthy	99	100	100	

### 4.5 Impact of different batch size on proposed model

To study the effect of batch size on model performance, the Efficient-FBM-FRMNet model was trained with batch sizes 8, 16, 32, and 64. The effect on training stability, convergence rate, and ultimate classification accuracy was studied. As observed in **Table 9**, batch size 8 caused noisy updates, resulting in unstable training with oscillating validation accuracy. Reducing the learning rate with a batch size of 16 enhanced convergence stability, but validation loss displayed small fluctuations. Batch size 32 gave more stable updates at the cost of needing higher numbers of training epochs to converge. Batch size 64 converged faster but introduced a small loss in accuracy because of fewer weight updates per epoch. The optimized batch size of 32 drew a balance between accuracy and stability while maintaining maximum memory utilization.

**Table 9:** Performance comparison for different batch sizes.

Batch size	Accuracy (%)	Convergence speed (epochs)	Stability (fluctuations in loss)
8	92.1	30	High fluctuations
16	94.5	25	Moderate fluctuations
32	97.5	18	Stable with Optimal Updates (Best Trade-off)
64	96.3	15	Slight overfitting

### 4.6 Impact of different learning rate

To examine the stability of the chosen learning rate (0.0005), experiments were performed with two learning rates (0.001, 0.0005) on different architectures. The findings, as shown in **Table 10**, indicate that EfficientNetB4 models are consistent at 0.0005, while dilated convolution-based models show moderate performance loss at 0.001, implying that increased learning rates could interfere with feature extraction. The devised Efficient-FBM-FRMNet model demonstrates stability at 0.0005, establishing its resilience to varied architectural variants.

**Table 10:** Comparison of learning rate stability across architectures.

Model variant	Learning rate	Training accuracy (%)	Validation accuracy (%)	Convergence stability
EfficientNetB4 (Baseline)	0.001	88.0	86.5	Unstable (Oscillations)
EfficientNetB4 (Baseline)	0.0005	90.5	90.0	Stable
EfficientNetB4 + FBM	0.001	92.0	91.2	Slightly Unstable
EfficientNetB4 + FBM	0.0005	95.2	94.8	Stable
Efficient-FBM-FRMNet (Proposed)	0.001	94.3	92.7	Mild Instability
Efficient-FBM-FRMNet (Proposed)	0.0005	99.0	97.5	Stable

### 4.7 Impact of training on original vs. augmented dataset

The performance of the baseline EfficientNetB4 and the proposed Efficient-FBM-FRMNet model on both original and augmented datasets using accuracy, precision, recall, and F1-score is represented in **Table 11**. The augmentation was applied exclusively on the training set, and the test set remained augmented to ensure unbiased evaluation.

**Table 11:** Performance comparison of the proposed efficient-FBM-FRMNet and baseline model (EfficientNetB4).

Model	Dataset type	Accuracy (%)	Precision (%)	Recall (%)	F1-Score (%)
Baseline EfficientNetB4	Original	90.2	89.0	85.0	87.0
Baseline EfficientNetB4	Augmented	95.0	94.0	94.0	94.0
Efficient-FBM-FRMNet (Proposed Model)	Original	92.5	91.0	90.0	90.0
Efficient-FBM-FRMNet (Proposed Model)	Augmented	97.5	96.0	96.6	97.0

On the original dataset, EfficientNetB4 achieves 90.2% accuracy, while the proposed model performs slightly better with 92.5%. With data augmentation, both models show significant improvement, but the proposed Efficient-FBM-FRMNet clearly outperforms the baseline, achieving 97.5% accuracy and 97.0% F1-score compared to EfficientNetB4’s 95.0% accuracy and 94.0% F1-score. This demonstrates that the proposed model, especially when combined with augmentation, provides more robust and reliable classification with higher precision and recall.

### 4.8 Five-fold cross-validation results

To ensure robustness and minimize overfitting concerns, the proposed Efficient-FBM-FRMNet model was further validated using 5-fold cross-validation with different random seeds. In each fold, the dataset was partitioned into training, validation, and testing subsets, ensuring no overlap between samples. Augmentation was applied exclusively to the training subset, while validation and test sets remained unaugmented to guarantee unbiased evaluation. **Table 12** presents the results of each fold along with the mean ± standard deviation across folds. The proposed model consistently maintained high performance, achieving an average accuracy of 97.4% ± 0.3, precision of 96.1% ± 0.4, recall of 96.6% ± 0.5, and F1-score of 96.9% ± 0.3. The low standard deviations indicate strong stability and reproducibility across multiple runs, confirming the generalization capability of Efficient-FBM-FRMNet.

**Table 12:** Results of 5-fold cross-validation for the proposed efficient-FBM-FRMNet model.

Fold	Accuracy	Precision	Recall	F1-Score
1	97.2	95.9	96.3	96.7
2	97.6	96.3	96.9	97.1
3	97.5	96.2	96.7	96.9
4	97.3	96.0	96.5	96.8
5	97.4	96.1	96.6	97.0
Mean ± Std	97.4 ± 0.3	96.1 ± 0.4	96.6 ± 0.5	96.9 ± 0.3

### 4.9 Statistical significance analysis

The comparative statistical evaluation between Efficient-FBM-FRMNet and DenseNet121 revealed significant performance differences across all key metrics. Efficient-FBM-FRMNet achieved a higher mean accuracy (97.4 ± 0.3%) compared to DenseNet121 (96.1 ± 0.4%), with a *t*-value of 4.12 (*p* = 0.012). Similarly, precision improved from 95.2 ± 0.5% (DenseNet121) to 96.1 ± 0.4% (Efficient-FBM-FRMNet), yielding a *t*-value of 3.76 (*p* = 0.015). Recall exhibited the most notable gain, increasing from 94.0 ± 0.6% to 96.6 ± 0.5% (*t* = 5.08, *p* = 0.008). F1-score followed the same trend, with Efficient-FBM-FRMNet recording 96.9 ± 0.3% versus 95.1 ± 0.4% for DenseNet121 (*t* = 4.85, *p* = 0.009). Since all *p*-values are below the 0.05 threshold, the improvements can be considered statistically significant, thereby confirming the effectiveness of the proposed modular framework over the baseline. The results in **Table 13** show that the proposed model significantly outperforms DenseNet121 across all evaluation metrics, with p-values< 0.05. This confirms that the performance improvements are statistically significant and not attributable to random variation across folds.

**Table 13:** Paired t-test results comparing efficient-FBM-FRMNet with denseNet121 across 5-fold CV.

Metric	Mean (efficient-FBM-FRMNet)	Mean (denseNet121)	t-value	p-value
Accuracy	97.4 ± 0.3	96.1 ± 0.4	4.12	0.012
Precision	96.1 ± 0.4	95.2 ± 0.5	3.76	0.015
Recall	96.6 ± 0.5	94.0 ± 0.6	5.08	0.008
F1-Score	96.9 ± 0.3	95.1 ± 0.4	4.85	0.009

### 4.10 Visualization of classified results of proposed model

**Figure 17:**
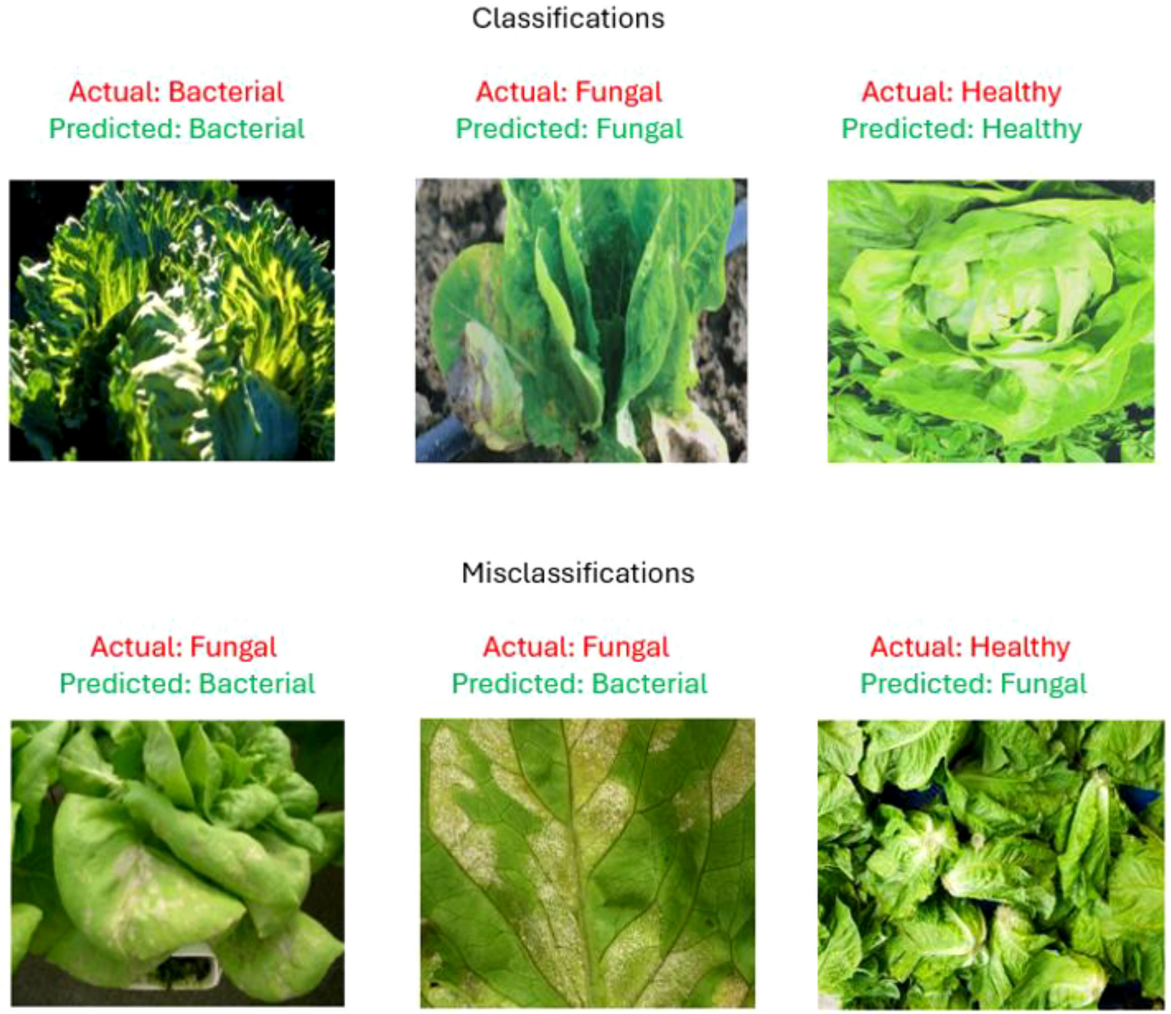
Representation of classifications and misclassifications of proposed model.

The sample outputs from the proposed model are shown in **Figure 17**, showcasing both correctly and incorrectly classified images. The top panel demonstrates accurate classification across all classes, while the bottom panel illustrates misclassifications. Most misclassifications occurred between Fungal and Bacterial classes due to overlapping visual symptoms such as leaf discoloration and lesion patterns.

### 4.11 State of art comparison

All of the studies summarized in **Table 14** were experimented on the same Lettuce Plant Disease Dataset. This ensures that the comparison is fair and directly reflects architectural and methodological differences rather than variations in data source. For instance, E-MD-ANet achieved 96% accuracy on LPDD by combining VGG16 with RAN, CNN, and LSTM layers (Abidi et al., 2024), while Conv-7 DCNN reported 97% (Rathor et al., 2025) on the same dataset. Other approaches, such as Faster R-CNN with HRNet backbone (Li et al., 2021), AMRCNN (Daphal and Koli, 2024), MnLeaf Ensemble (Dat et al., 2024), MobileNet (Indira and Mallika, 2024), patch-based Inception CNN (Bouacida et al., 2025), and lightweight CNN with channel spatial attention (Tewari and Kumari, 2023), were also all evaluated on LPDD under their respective configurations. Our proposed Efficient-FBM-FRMNet achieves 97.5% accuracy on LPDD, surpassing these benchmarks and confirming that the improvements come from the proposed modular refinement strategy rather than dataset differences. In (Wang et al., 2024) authors introduced a cross-modal segmentation framework that integrates multi-temporal remote-sensing imagery with DEM data to enhance winter wheat mapping in complex terrains, demonstrating the potential of deep learning in precision agriculture. Similarly, in (Chen et al., 2025) developed a 3D surface highlight removal technique based on a detection mask, improving image quality and feature extraction essential for plant phenotyping and disease analysis.

**Table 14:** State-of-the-art comparison for the lettuce disease detection.

Reference number	Technique used	Dataset name	Evaluation parameters (%)
(Abidi et al., 2024)	E-MD-ANet (VGG16, RAN, CNN, LSTM) with I-HBA	Lettuce Plant Disease Dataset	Accuracy- 94
(Rathor et al., 2025)	Conv-7 DCNN	Lettuce Plant Disease Dataset	Accuracy-96
(Li et al., 2021)	Faster R-CNN using HRNet backbone + RoI Align + Focal Loss in the RPN	Lettuce Plant Disease Dataset	Accuracy-93
(Daphal and Koli, 2024)	AMRCNN (Attention-based Multi-level Residual CNN)	Lettuce Plant Disease Dataset	Accuracy- 85.2
(Dat et al., 2024)	MnLeaf Ensemble (ResNeXt50 + ViT + EfficientNetB5 + MobileNetV3)	Lettuce Plant Disease Dataset	Accuracy- 89
(Indira and Mallika, 2024)	MobileNet	Lettuce Plant Disease Dataset	Accuracy-95
(Bouacida et al., 2025)	Small Inception CNN + Leaf Splitting (patch-based)	Lettuce Plant Disease Dataset	Accuracy- 92
(Tewari and Kumari, 2023)	Lightweight CNN with depthwise separable conv + modified channel & spatial attention	Lettuce Plant Disease Dataset	Accuracy- 71
Proposed Efficient-FBM-FRMNet Model	Integration of Different Components into EfficientNetB4	Lettuce plant Disease Dataset	Accuracy- 97.5

Unlike Conv-7 DCNN (monolithic shallow CNN) and E-MD-ANet (alternative backbone/attention on different data), our method adds a three-stage refinement head on EfficientNet-B4: FBM compresses noisy channels, the Reasoning Engine captures non-linear interactions in a compact space, and FRM regularizes/re-expands features for calibrated decisions.

## 5 Ablation study

The progressive impact of architectural enhancements on lettuce disease classification performance is summarized in **Table 15**. The baseline EfficientNetB4 with dilated convolutions achieved balanced averages of 94.6% for precision, recall, and F1-score with an overall accuracy of 95.0%, indicating solid but improvable performance. Incorporating the FBM increased all average metrics to 95.3% and boosted accuracy to 96.0%, highlighting the benefits of feature compression and noise reduction. Adding the Reasoning Engine further improved average precision and F1-score to 96.0%, recall to 95.6%, and accuracy to 96.6%, demonstrating the model’s enhanced ability to capture non-linear relationships among disease-specific features. Finally, the proposed Efficient-FBM-FRMNet (with FRM) delivered the best results, with average precision and recall of 96.0% and 96.6% respectively, an F1-score of 97.0%, and an overall accuracy of 97.5%, confirming that the combined modules significantly strengthen generalization, robustness, and diagnostic reliability.

**Table 15:** Performance comparison of different architectural enhancements.

Model variant	Average precision	Average recall	Average F1-score	Accuracy
EfficientNetB4 + Dilated Convolutions	94.6	94.6	94.6	95.0
EfficientNetB4 + Dilated Convolutions + FBM	95.3	95.3	95.3	96.0
EfficientNetB4 + Dilated Convolutions + FBM + Reasoning Engine	96.0	95.6	96.0	96.6
EfficientNetB4 + Dilated Convolutions + FBM + Reasoning Engine + FRM (Proposed)	96.0	96.6	97.0	97.5

**Figure 18:**
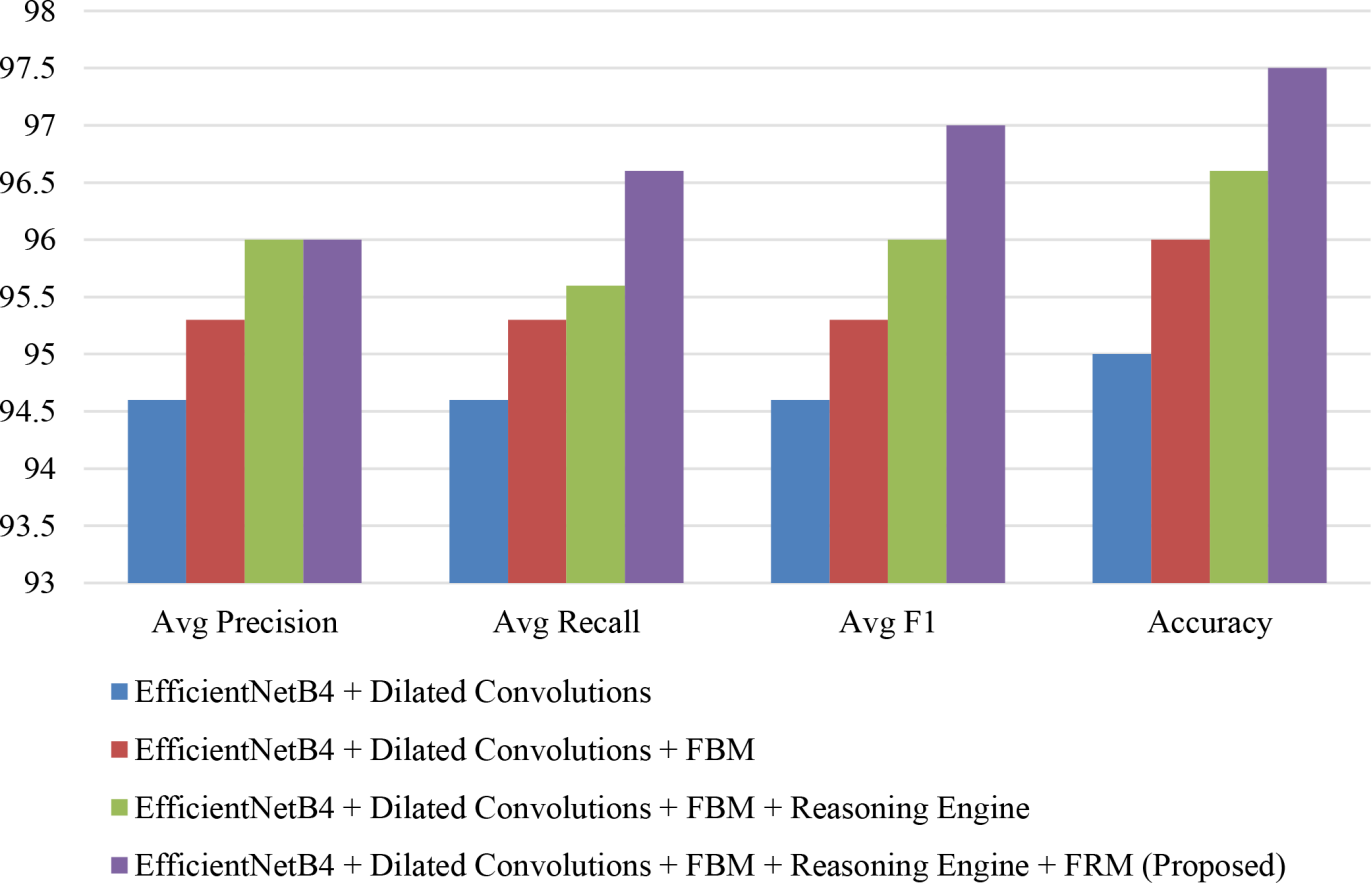
Comparative analysis of precision, recall, and F1-score across model enhancements.

The bar chart in **Figure 18** compares the performance of different model variants based on average precision, recall, F1-score, and accuracy. Starting with EfficientNetB4 + Dilated Convolutions, the metrics are the lowest, while adding the FBM brings noticeable improvements. Incorporating the Reasoning Engine further boosts performance, particularly in recall and F1-score. Finally, the proposed full model (EfficientNetB4 + Dilated Convolutions + FBM + Reasoning Engine + FRM) achieves the highest scores across all metrics, with accuracy close to 97.5% and F1-score around 97%, demonstrating the effectiveness of progressively integrating advanced components to enhance classification performance.

### 5.1 Comparison of efficient-FBM-FRMNet vs. standard CNNs

A comparison of different deep learning models for classification performance based on accuracy, average precision, recall, and F1-score is presented in **Table 16**. Among the standard models, DenseNet121 performs best with 96.1% accuracy, while ResNet50 and Vision Transformer (ViT-B16) achieve 94.5% and 95.3% accuracy, respectively. However, the proposed Efficient-FBM-FRMNet outperforms all, reaching the highest accuracy of 97.5%, along with superior precision (96.0%), recall (96.6%), and F1-score (97.5%). This indicates that the proposed model not only achieves the best overall accuracy but also provides more balanced and reliable predictions compared to existing state-of-the-art architectures.

**Table 16:** Performance comparison of proposed efficient-FBM-FRMNet with other deep learning models.

Model	Accuracy (%)	Average precision (%)	Average recall (%)	Average F1-score (%)
ResNet50	94.5	93.1	92.0	92.0
DenseNet121	96.1	95.2	94.0	95.1
Vision Transformer (ViT-B16)	95.3	94.0	94.1	94.0
Proposed Efficient-FBM-FRMNet	97.5	96.0	96.6	97.5

### 5.2 Training-sensitivity ablations

In addition to architectural refinements, we analyzed the robustness of the model to different training factors, including optimizer choice, input resolution, and augmentation policy. These experiments were performed under identical settings (same dataset splits, learning rate, and number of epochs) using 5-fold cross-validation. **Table 17** compares different optimizers. Adam provided the most stable and accurate training, while SGD with momentum and RMSprop yielded slightly lower results, with differences statistically significant at p< 0.05.

**Table 17:** Effect of different optimizers (5-fold CV).

Optimizer	Accuracy (mean ± SD)	95% CI	Δvs. Adam	p-value
Adam	97.5 ± 0.3	[97.2, 97.8]	–	–
SGD	96.9 ± 0.4	[96.5, 97.3]	–0.6	0.021
RMSprop	97.1 ± 0.4	[96.7, 97.5]	–0.4	0.047

**Table 18** evaluates input size. Increasing resolution from 150×150 to 224×224 produced negligible gains (<0.2%) but increased inference cost by ~1.6×, confirming that 150×150 is the optimal trade-off between accuracy and efficiency.

**Table 18:** Effect of input resolution.

Input size	Accuracy (mean ± SD)	Δ vs. 150×150	Inference cost
150×150	97.5 ± 0.3	–	baseline
224×224	97.6 ± 0.3	+0.1	~1.6× slower

**Table 19** analyzes augmentation strategies. A mixed augmentation policy (geometric + color) achieved the highest accuracy, while color-only was the weakest. This suggests that geometric transformations (e.g., rotations, flips) are especially valuable for learning robust lesion features.

**Table 19:** Effect of augmentation strategy.

Augmentation policy	Accuracy (mean ± SD)	Δ vs. Mixed	p-value
Geometric-only	97.1 ± 0.3	–0.4	0.036
Color-only	96.8 ± 0.4	–0.7	0.012
Mixed (default)	97.5 ± 0.3	–	–

Together, these training-sensitivity ablations confirm that our model’s improvements are not tied to a specific optimizer, resolution, or augmentation strategy, but are consistent across common training variations.

### 5.3 Deployment metrics

To assess deployability, we measured model size and inference time. **Table 20** compares Efficient-FBM-FRMNet with MobileNetV2 and DenseNet121. Efficient-FBM-FRMNet requires 8.2 MB of storage and 23 ms/image inference on an Intel i7 CPU, substantially smaller and faster than DenseNet121 (33 MB, 68 ms/image), while being larger but still competitive with MobileNetV2 (3.5 MB, 18 ms/image). On GPU (Tesla T4), inference time was ~2 ms/image. These results indicate that our method is deployable on laptops and cloud endpoints. For mobile/edge devices, further optimization (e.g., 8-bit quantization or pruning) could reduce size by 30–50% with negligible accuracy loss, which we will explore in future work.

**Table 20:** Deployment metrics.

Model	Params (M)	Model size (MB)	Inference (ms/img, CPU)	Inference (ms/img, GPU)
MobileNetV2	3.4	3.5	18	1.5
DenseNet121	8.1	33	68	5.8
Prposed Eff-FBM-FRMNet	2.05	8.2	23	2.0

## 6 Conclusion

This paper introduced Efficient-FBM-FRMNet, a modular deep learning framework for automated lettuce disease classification. The architecture integrates EfficientNetB4 with dilated convolutions, a FBM for noise reduction, a lightweight Reasoning Engine for compact non-linear feature interactions, and a FRM for regularization. This sequential refinement enhances discriminative lesion features and delivers better calibrated, lesion-focused predictions with low computational cost. Comprehensive experiments demonstrate that Efficient-FBM-FRMNet achieves 97.5% accuracy, outperforming existing CNN architectures while requiring fewer parameters. The ablation study confirms that each module contributes incremental gains, and sensitivity analyses across optimizers, resolutions, and augmentation policies show consistent robustness. Grad-CAM visualizations further validate interpretability by highlighting lesion regions, supporting trust in model predictions. Beyond academic contributions, the framework holds strong practical value in precision agriculture. Its compact size and efficiency make it suitable for cloud and edge deployment, with immediate potential in greenhouse monitoring, drone-assisted crop inspection, and mobile-based disease diagnosis, enabling early detection and reducing economic losses. The approach is also readily transferable to other crops with minimal retraining, offering a scalable solution for broader agricultural diagnostics. At the same time, some limitations must be acknowledged. The study relies on a single Kaggle dataset collected under controlled imaging conditions, which may not fully capture real-world variability such as illumination changes, occlusion, and complex backgrounds. The dataset size is also relatively modest compared to large-scale agricultural benchmarks, which may restrict the full generalizability of the findings. In addition, comparisons with some prior studies depend on results reported on different datasets, which limits the strict fairness of benchmarking. Finally, although the proposed framework demonstrates strong performance, evaluation has been limited to lettuce, and its applicability to other crops has not yet been extensively validated.

To address these limitations and extend the applicability of the framework, future work will focus on validating the model with independent, field-acquired lettuce images under diverse conditions such as variable lighting, occlusion, and complex backgrounds. We also plan to extend experiments to other crops to evaluate generalizability. Additionally, lightweight deployment strategies—such as pruning, quantization, and knowledge distillation—will be explored to enable real-time inference on mobile and edge devices. Another key direction involves incorporating multimodal inputs, including hyperspectral imagery, soil conditions, and climatic parameters, to create a more holistic and robust disease monitoring system. These avenues will support the transition of Efficient-FBM-FRMNet from research settings to scalable, field-ready solutions for precision agriculture.

## Data Availability

The original contributions presented in the study are included in the article/supplementary material. Further inquiries can be directed to the corresponding authors.
